# Treatment of Acute Coronary Syndrome by Telemedically Supported Paramedics Compared With Physician-Based Treatment: A Prospective, Interventional, Multicenter Trial

**DOI:** 10.2196/jmir.6358

**Published:** 2016-12-01

**Authors:** Jörg C Brokmann, Clemens Conrad, Rolf Rossaint, Sebastian Bergrath, Stefan K Beckers, Miriam Tamm, Michael Czaplik, Frederik Hirsch

**Affiliations:** ^1^Emergency DepartmentRheinisch-Westfälische Technische HochschuleUniversity Hospital RWTH AachenAachenGermany; ^2^Department of AnaesthesiologyRheinisch-Westfälische Technische HochschuleUniversity Hospital RWTH AachenAachenGermany; ^3^Department of Medical StatisticsRheinisch-Westfälische Technische HochschuleUniversity Hospital RWTH AachenAachenGermany

**Keywords:** acute coronary syndrome, prehospital emergency care, telemedicine, telehealth, myocardial infarction

## Abstract

**Background:**

Prehospital treatment of acute coronary syndrome (ACS) in German emergency medical services (EMSs) is reserved for EMS physicians due to legal issues.

**Objective:**

The objective of this prospective, interventional, multicenter trial was to evaluate the quality of telemedically-delegated therapy and the possible complications in patients with ACS.

**Methods:**

After approval by the ethics committee and trial registration, a one-year study phase was started in August 2012 with 5 ambulances, telemedically equipped and staffed with paramedics, in 4 German EMS districts. The paramedics could contact an EMS-physician–staffed telemedicine center. After initiation of an audio connection, real-time data transmission was automatically established. If required, 12-lead electrocardiogram (ECG) and still pictures could be sent. Video was streamed from inside each ambulance. All drugs, including opioids, were delegated to the paramedics based on standardized, predefined algorithms. To compare telemedically-delegated medication and treatment in ACS cases with regular EMS missions, a matched pair analysis with historical controls was performed.

**Results:**

Teleconsultation was performed on 150 patients having a cardiovascular emergency. In 39 cases, teleconsultation was started due to suspected ACS. No case had a medical complication. Correct handling of 12-lead ECG was performed equally between the groups (study group, n=38 vs control group, n=39, *P*>.99). There were no differences in correct handling of intravenous administration of acetylsalicylic acid, heparin, or morphine between both the groups (study group vs control group): acetylsalicylic acid, n=31 vs n=33, *P*=.73; unfractionated heparin, n=34 vs n=33, *P*>.99; morphine, n=29 vs n=27, *P*=.50. The correct handling of oxygen administration was significantly higher in the study group (n=29 vs n=18, *P*=.007).

**Conclusions:**

Telemedical delegation of guideline conform medication and therapy by paramedics in patients with ACS and was found to be feasible and safe. The quality of guideline-adherent therapy was not significantly different in both the groups except for the correct administration of oxygen, which was significantly higher in the study group.

**Trial Registration:**

Clinicaltrials.gov NCT01644006; http://clinicaltrials.gov/ct2/show/NCT01644006 (Archived by WebCite at http://www.webcitation.org/6mPam3eDy).

## Introduction

The in-hospital mortality rate of ST Segment Elevation Myocardial Infarction (STEMI) could be significantly reduced by the modern reperfusion therapy and improved secondary prophylaxis. However, the overall mortality remains unchanged because two-thirds of deaths occur during the prehospital phase—usually due to lethal arrhythmias that are triggered by ischemic events [1]. Therefore, an appropriate therapy must be initiated in the early prehospital phase, and the time of admission must be as small as possible to improve survival. This is also important in non–STEMI-acute coronary syndrome (NSTE-ACS) as the incidence of NSTE-ACS rises further [2,3].

Telemedical support for diagnosis and therapy in acute coronary syndromes (ACSs) has been established for quite some time in emergency medical services (EMS). Several telemetry projects have demonstrated feasibility [4-7] in the transmission of a 12-lead electrogram (ECG) or its images to a cardiologist. The cardiologist receiver can then consecutively assess the potential to improve survival and outcome in STEMI [7]. It even has been shown that an artificial neural network could predict STEMI and the need of acute percutaneous coronary intervention (PCI) in ambulance ECGs [8]. However, all of these studies assessed the impact of 12-lead ECG transmission on in-hospital parameters and outcome in STEMI patients. Evidence regarding telemedical concepts in non-STEMI ACS patients is mostly lacking.

Germany uses a two-tiered EMS system with paramedic-staffed ambulances and additional EMS with physician response units. Like some other European countries, only an EMS physician can administer the medication required in ACS. Except for nitrates, paramedics cannot dispense drugs. In cases where primary dispatch was merely a paramedic-staffed ambulance and the EMS physician was notified later, the time to arrival and thus the administration of medication may be unacceptably long. This effect is exacerbated in more rural areas and by an occasional lack of physician-staffed EMS [9,10].

Against this background, a mobile telemedicine system was developed during a first research project from 2007 to 2010. Two comprehensive simulation studies demonstrated that real-time telemedical support by experienced remote EMS physicians leads to improved quality of care in STEMI. Even telemedically supported paramedics were able to handle emergency care on their own with a comparable performance compared with on-scene physicians [11,12]. The main findings during the project, preceding to the one described here, were the feasibility of prehospital teleconsultation in general and the improvement of data transmission in acute stroke [13,14]. This succeeding research project allowed further technical and organizational development. The telemedical equipment was made more practicable and paramedic-staffed ambulances were equipped with this system. Thus, the concept allowed medications to be given by paramedics in ACS when supervised by an experienced EMS physician—the so-called tele-EMS physician. In this prospective, interventional trial, we assessed the quality of prehospital emergency care in patients suffering from ACS (measured by adherence to international guidelines) when using a telemedically supported EMS. Using matched pairs, we compared our findings with a comparable historical control group treated by our regular physician-based EMS.

## Methods

This prospective, interventional, multicenter trial was conducted from August 1, 2012 to July 31, 2013 within the research project “TemRas” (telemedical rescue assistance system).

### Study Setting

In 4 different EMS districts in Germany, 5 paramedic-staffed ambulances were stepwise equipped with a multifunctional telemedicine system. Three of the 4 EMS districts were rural and one was urban (Table 1). As described elsewhere, all participating EMS physicians in the function of a “tele-EMS physician” were trained prior to the start of the intervention phase [15]. All participating tele-EMS physicians had a minimum experience of 3 years in anesthesia and critical care as well as broad experience as an EMS physician. The paramedics involved (N=178) ran through a standardized eight-hour training program before the project started. The goal was to learn the use of the technical system, the medical concept of teleconsultation including indications for teleconsultation (eg, ACS), and communication skills [15].

**Table 1:** Demographics and structure of the participating emergency medical service (EMS) districts.

Demographics		Aachen (urban)	Heinsberg (rural)	Dueren (rural)	Euskirchen (rural)
Population		248,137	256,546	267,712	190,591
Area (km^2^)		160.8	628.0	941.4	1248.7
**Ambulances**	
	24-h Ambulances (telemedically equipped^a^)	6 (2)	7 (1)	11 (1)	9 (1)
	Daytime ambulances	2	3	2	2
	EMS^b^ physician units	2+1^c^	4	4+1^d^	3
	Ambulance emergency missions/year	22,984	14,346	20,302	15,108
	EMS physician missions/year	7898	7786	9057	5317
**Hospitals**		4	4	5	3
	Stroke units	1	1	1	1
	Level 1 trauma center	1	-	-	-
	24-h Cardiac catheterization laboratory	1	1	1	1
	Number of cases (n)	18	6	3	12

^a^telemedically equipped for 24 hours, operation of teleconsultation center: 7:30 am-4:30 pm.

^b^EMS: emergency medical service.

^c^additional EMS physician at home can be picked up by the fire department.

^d^daytime EMS physician unit: 7 am-4 pm.

### Trial Registration and Ethical Issues

The trial was registered before the intervention phase (clinicaltrials.gov NCT01644006). The study was approved by the local ethics committee (University Hospital Rheinisch-Westfälische Technische Hochschule Aachen, Germany, registration number EK 191/11). The written informed consent was waived by the ethics committee due to the emergency setting; however, all alert patients had to give verbal approval to data and video transmission before teleconsultation. Data from the preintervention phase was analyzed retrospectively via prospectively planned outcomes of the intervention phase. Multimedia Appendix 1 shows the trial protocol.

### Technical System

An MRx monitor-defibrillator unit (Philips Healthcare, Andover, MA, USA) connected to a portable data transmission unit (peeqBox, P3 communications, Aachen, Germany) was used by the paramedics to establish parallelized and encrypted data and audio connection via different mobile networks. The audio channel was accomplished with 2 headsets (Voyager Pro HD, Plantronics, Santa Cruz, CA, USA) connected via Bluetooth to the mobile transmission unit. Real-time vital data transmission (numerical values and curves, Einthoven leads I-III continuously) occurred automatically after initiation of a teleconsultation. If required, 12-lead ECGs could be transmitted. Still pictures could be sent using a Bluetooth connection between a mobile phone (HTC Sensation XE, High Tech Computer Corporation, Taoyuan, Taiwan) and the data transmission unit. The tele-EMS physician could start a video streaming from a ceiling camera of the ambulance when the patient was inside in the ambulance. On the telemedical workstation, real-time vital data was displayed using the IntelliVue Information Centre (Philips Healthcare, Boeblingen, Germany), and 12-lead-ECGs were displayed on the HeartStart Telemedicine Viewer (Philips Healthcare, Andover, MA, USA). All other software components in the telemedicine center were specifically developed as part of the research project. In cases of ACS, an established algorithm could be displayed on the workstation to support the tele-EMS physician in guideline-adherent therapy recommendations including the steps needed to diagnose and dose medication as recommended by national and international guidelines. A detailed technical description of the telemedicine system was already described elsewhere [16].

### Interventions

The decision to initiate a teleconsultation was solely made by the on-scene paramedics. All participating EMS districts ran their own dispatch centers. Local protocols for ambulance and EMS physician units’ alarm were not changed before or within the project period. The telemedicine system was used in addition to the regular EMS system. If the EMS dispatch centers suspected ACS, then an EMS physician response unit was sent in accordance to the special provisions of law for paramedic-staffed ambulance. Therefore, only cases where the initial notification was different or cases that bridged the arrival of an EMS physician unit could be managed with telemedical care. The additional telemedicine service was available during workdays from 7:30 am to 4:30 pm during the first 4 months of the intervention phase and from 7:30 am to 8:00 pm for the rest of the trial phase due to restricted funding. No teleconsultation system existed during the preintervention period.

### Outcomes and Data Sources

The following data sources were used: the teleconsultation protocols, paper-based EMS protocols of the participating EMS stations, and data of the local EMS dispatch centers. The quality of prehospital care of ACS based on national and international published guidelines [17-21] for non–STEMI-ACS and STEMI was analyzed as the primary outcome measure. Guideline adherence was measured by correct handling of the following measures: 12-lead ECG completed; administration of acetylsalicylic acid≥250mg iv, independent of long-term therapy of the patient; administration of heparin 70 IU/kg iv, maximum 5000 IU, but waiver in the presence of effective anticoagulation in patient’s long-term therapy; administration of morphine if numerical rating scale (NRS)≥3 (repeatedly 3-5 mg iv until NRS≤3); and administration of oxygen if oxygen saturation≤95%. Treatment was not carried out in cases of any allergies or contraindications. If so, this was measured as correct handling.

A standardized report for any potential problems or adverse events was completed daily by the tele-EMS physician in charge. The adverse events were defined as follows: respiratory or circulatory insufficiency due to administered medications with a need for intervention or allergic reaction due to administered medications. Multimedia Appendix 2 shows the original dataset for further statistical evaluation.

### Sample Size

The trial had a restricted funding period of one year. Because this was the first study of prehospital telemedically-delegated diagnosis and therapy including medication in patients with ACS in this setting, a power analysis and formal sample size calculation could not be performed before this pilot study. A possible sample size was estimated by an expert group and documented in the trial registration. The anticipated enrolment of the sample size was N=180.

### Inclusion and Exclusion Criteria

**Figure 1:**
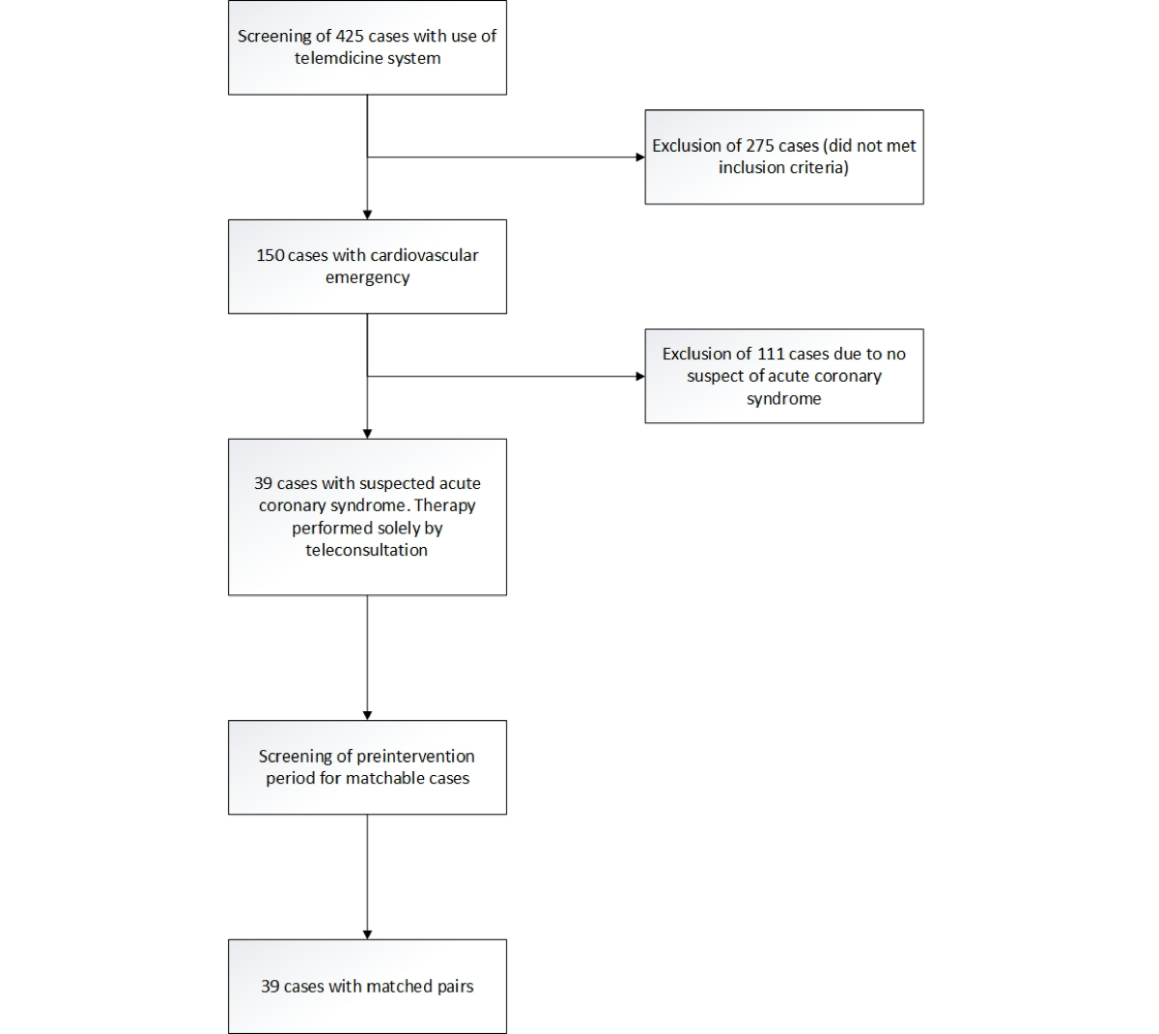
Study flowchart.

Patients were included if ACS was diagnosed, verbal consent for teleconsultation was obtained, and were aged ≥18 years. Patients who refused consent to teleconsultation or had no suspected ACS were excluded. Figure 1 shows the study flowchart.

### Matched Pairs

The matched pairs were searched from a historical preintervention period (April 1, 2011 to March 31, 2012) that was one consecutive year prior to the first training lesson. Matching was done using predefined criteria (Table 2) and the nearest-neighbour matching method for all cases that fulfilled the matching criteria.

**Table 2:** Matching criteria.

Matching criteria	Matching categories
EMS^a^ district	Study group cases and control group cases had to be from same district
Date of emergency mission	During the historical control period, the same month and calendar day was used as starting point for the backward and forward search; the first case that fulfilled the matching criteria listed below was included
Patient’s age	Same age ± 10 years
Sex	Female
	Male
Symptoms	Chest pain (typically)
	No chest pain (atypically), but other symptoms (ie, nausea, vomiting)
	No symptoms

^a^EMS: emergency medical service.

### Statistical Methods

Categorical parameters were compared using 4 field contingency tables with McNemar’s Test. All statistical analyses were performed using SAS 9.4 (SAS Institute Inc). *P* values <.05 were considered to be significant. Multimedia Appendix 3 shows the statistical evaluation.

## Results

Overall, 425 emergency teleconsultation cases were performed with 150 patients having a cardiovascular emergency. The demographic data (Multimedia Appendix 4) was spread equally between the study group and the control group. In each group, 14 patients were female (36%, 14/39), the rest were male. In 39 cases, teleconsultation was started due to suspected ACS. In the described cases, no adverse events were detected. Correct handling of 12-lead ECG was performed equally between the groups (study group, n=38 vs control group, n=39; *P*>.99). There was no significant difference in correct handling of intravenous administration of acetylsalicylic acid (n=38), heparin (n=38), or morphine (n=29) between both groups (study group vs control group): acetylsalicylic acid, n=31 vs n=33, *P*=.73; unfractionated heparin, n=34 vs n=33, *P*>.99; and morphine, n=29 vs n=27 *P*=.50. The correct handling of oxygen differed significantly between the groups (n=34). In the study group it was performed correctly for n=29 and for n=18 in the control group (*P*=.007). Multimedia Appendix 5 gives a detailed view of the correct or incorrect handling of each measure in each of the participating districts. Table 3 summarizes the above mentioned results.

**Table 3:** Summary of results of correct handling of measures.

Measures	n	Study group	Control group	*P* value
12-lead ECG^a^	39	38	39	>.99
Acetylsalicylic acid	38	31	33	.73
Heparin	38	34	33	>.99
Morphine	29	29	27	.50
Oxygen	34	29	18	.007

^a^ECG: electrocardiogram.

## Discussion

### Principal Findings

To the best of our knowledge, this is the first study providing prehospital diagnosis and emergency care for ACS that was completely delivered by telemedically supported paramedics. The telemedical concept is feasible, and guideline adherence was at least comparable with on-scene physician care with improved guideline adherence regarding oxygen administration. The feasibility and improvements on outcome by wireless transmission of ECGs to specialists have already been shown in STEMI [4,5,7]. As demonstrated here, both groups handled the administration of the 12-lead ECG correctly. Due to legal restrictions in Germany, the full administration of required medication in patients with ACS administered by paramedics is completely new to the field of prehospital care. The required medication for each patient in the study group was delegated by the tele-EMS physician. Between groups, there were no significant differences in the correct handling of intravenous administration of acetylsalicylic acid, heparin, or morphine. It was already shown that telemedically supported administration of analgesics by paramedics is feasible and secure [22]. Regarding the pain therapy in ACS described here, both groups could improve the correct handling of morphine. We noted that morphine should be administered when NRS was ≥3 (repeatedly 3-5 mg iv). Eventually, morphine was given in both groups even if NRS was not ≥3 or pain reduction was not achieved in a sufficient extent.

In the study group, the oxygen administration was performed significantly more correctly than in the control group. This may be because the tele-EMS physician had a predefined algorithm for correct treatment of ACS at any time in digital form that was integrated in the documentation system. As this algorithm stated, oxygen had to be applied when the blood saturation was ≤95%. We assume that oxygen was applied in the control group all too often, even if the oxygen saturation was above 95%. Therefore, the differences are explained by improved guideline adherence.

Neither the telemedicine group nor the control group had any medical complication. Due to the small sample size, a definitive statement regarding the safety of complete prehospital ACS therapy by paramedics and tele-EMS physicians cannot be given. However, these findings support the thesis that telemedically supported prehospital diagnosis and therapy on the whole is a safe procedure, although the process was completely new for the participating physicians as well as for the paramedics. In comparison with ground- or helicopter-based EMS physician operations [23], this concept allows for a spatially unrestricted emergency concept with fewer resources. At this point it has to be mentioned that, to our knowledge, most studies with 12-lead-ECG transmission represent rather small sample sizes. Therefore, a large randomized controlled trial is needed.

No case had major technical problems. Although the technical performance of the telemedicine system has not yet been evaluated in detail, no consultation had to be terminated due to technical problems.

### Limitations

This pilot study had no formal sample size calculation and power analysis. Therefore, all results have to be interpreted against this background. This was the first pilot study to evaluate the concept of telemedically-assisted prehospital therapy in ACS to allow safe, effective, and guideline-adherent therapy. On the basis of these pilot results, a confirmatory trial with a calculated sample size would be meaningful. However, it cannot be definitely stated that telemedically assisted treatment is as effective as treatment with an on-site EMS physician. Due to medical privacy policy in Germany, we could not obtain in-hospital follow-up data and therefore could not highlight outcome parameters such as in-hospital time intervals in STEMI (contact to balloon time, arrival to balloon time), the rate of secondary transfer for PCI (rate of secondary transfer to a different facility for PCI), or laboratory analysis like troponin-levels.

### Conclusions

Prehospital diagnosis and treatment of ACS that was carried out by paramedics supported by tele-EMS physicians seems to be as safe and effective as the conventional procedure, assuming a holistic telemedical support system is used, as shown here. In fact, the supply and the adherence to national and international guidelines was better and not worse than in the conventional EMS physician-based service. Further trials with larger patient numbers and a randomized allocation are needed to confirm the findings of this pilot study.

## Multimedia Appendix 1

Trial protocol.

## Multimedia Appendix 2

Dataset.

## Multimedia Appendix 3

Statistical evaluation (raw dataset).

## Multimedia Appendix 4

Demographic data.

## Multimedia Appendix 5

Detail table.

## Abbreviations

ACS: acute coronary syndrome
ECG: electrocardiogram
EMS: emergency medical service
iv: intravenous
IU: international units
NRS: numeric rating scale
NSTE-ACS: non–STEMI-acute coronary syndrome
PCI: percutaneous coronary intervention
STEMI: ST elevation myocardial infarction
TemRas: Telemedical rescue assistance system
