# Supplementary material for: Treatment of Acute Coronary Syndrome by Telemedically Supported Paramedics Compared With Physician-Based Treatment: A Prospective, Interventional, Multicenter Trial
Source: J Med Internet Res. 2016 Dec 1;18(12):e314. doi: 10.2196/jmir.6358 (PMC5159613; doi:10.2196/jmir.6358)
Supplement: Supplementary file 1 [file jmir_v18i12e314_app1.pdf]

Prüfplan der Evaluationsphase von TemRas – Telemedizinisches Rettungsassistenzsystem

Inhaltsverzeichnis

1. Hintergrund....12
2. Studienziel....16
3. Probandenrekrutierung....19
4. Studiendurchführung....24
4.1 Methoden....24
Die Telekonsultation erfolgt zwischen einem Rettungswagenteam und einer Telenotarzt-Zentrale. Nach Eintreffen eines Notarztes kann dieser bei Bedarf zusätzlich in die Telekonsultation eingebunden werden oder die Telekonsultation wird nach Übergabe an den Notarzt vor Ort beendet....24
4.1.1 Telenotarzt-Zentrale....24
Die Telenotarzt-Zentrale wird mit erfahrenen Notärzten der Klinik für Anästhesiologie des Universitätsklinikums Aachens besetzt. Dazu werden ausschließlich Ärzte mit der Zusatzweiterbildung Notfallmedizin und mindestens Facharztstandard eingesetzt. Zudem verfügen die Ärzte über spezielle notfallmedizinische Zusatzqualifikationen in Reanimation, Herz-Kreislauf-Notfällen und Traumaversorgung (Advanced Life Support Course, Pre-Hospital Trauma Life Support). Ihr Einsatz in der Funktion als Telenotarzt erfolgt erst nach der oben erläuterten standardisierten Schulung....24
Die Telenotarzt-Zentrale wird räumlich bei der Berufsfeuerwehr Aachen eingerichtet. An zwei gleichartigen Bildschirmarbeitsplätzen können zwei Telenotärzte parallel Einsätze bearbeiten. Die Anzeige von Vitalparametern (Rhythmus-EKG, Sauerstoffsättigung, exspiratorisches CO_2, Blutdruck) und 12-Kanal-EKG erfolgt dabei nur durch dafür nach Medizinproduktegesetz zugelassene Systeme der Fa. Philips Healthcare (IntelliVue Information Center, Heartstart Telemedicine System). Zusätzlich zu diesen diagnostischen Instrumenten können übertragene Bilddateien und Videodateien angezeigt werden. Diese dienen ausdrücklich nicht der Diagnosefindung sondern nur zur Ergänzung der über die Sprachkommunikation ausgetauschten Gesprächsinhalte. Die Inhalte dieser Dateien müssen per Sprachkommunikation verifiziert werden. Zudem stehen dem Telenotarzt Handlungsempfehlungen (SOP), Leitlinien und Medikamentendatenbanken EDV-basiert zur Verfügung. In der Telenotarzt-Zentrale erfolgt eine softwarebasierte, standardisierte Dokumentation aller telemedizinisch betreuten Einsätze....24
Die Datenübertragung vom Rettungswagen bzw. der Einsatzstelle an die Telenotarzt-Zentrale sowie die bidirektionale Audioverbindung erfolgt stets verschlüsselt nach aktuellem Stand der Technik. Somit kann maximale Abhörsicherheit gewährleistet werden. Zur weiteren Datensicherheit werden alle Datenpakete mit einem elektronischen Schlüssel versehen. Bei Ankunft des Datenpaketes am Zielort (z.B. in der Telenotarzt-Zentrale) kann mit Hilfe dieses Schlüssels die Echtheit der Daten verifiziert

werden. Korrekt und nicht-korrekt übermittelte Daten können so differenziert werden und nur korrekt übertragene Daten kommen zur Anzeige. Die Speicherung dieser einsatzbezogenen Daten erfolgt auf speziell abgesicherten Servern. Dabei werden Videodaten grundsätzlich nicht abgespeichert. Nur die medizinische Projektleitung hat Zugriff auf die einsatzbezogenen Daten. 25
4.2.2 Rettungswagen 25
Insgesamt sechs Rettungswagen aus den fünf oben erwähnten Rettungsdienstbereichen werden telemedizinisch ausgestattet. Dabei werden zwei Systeme ergänzend zum Einsatz gebracht. Im Fahrzeug selbst kommt ein stationäres Übertragungssystem (P3 communications, Aachen) mit vier parallelen Datenkanälen aller deutschen Mobilfunkanbieter zum Einsatz. Dies bietet maximale Sicherstellung der Verfügbarkeit. Im Projekt Med-on-@ix konnte gezeigt werden, dass durch die parallele Verwendung von vier Datenkanälen die Wahrscheinlichkeit der Verfügbarkeit eines Datendienstes von 95% auf 99% steigt, im Vergleich zu einer kommerziellen Standardlösung mit einer einzelnen UMTS-Mobilfunkkarte. Zum mobilen Einsatz an einer Einsatzstelle (z.B. Wohnung des Patienten) wird eine kompakte, tragbare Datenübertragungseinheit (P3 communications, Aachen) eingesetzt, die ca. 1kg wiegt. Hiermit sind „nur" zwei parallele Datenkanäle, jedoch aller vier deutschen Mobilfunkanbieter, zeitgleich zu nutzen. Dieses stellt einen Kompromiss dar, da die mobile Einheit leicht und energiesparend sein muss, um eine einsatztaktisch ausreichend lange Akkulaufzeit zu haben. 25
Beide Systeme bieten folgende Funktionalitäten: 25
- Audioverbindung zwischen Rettungsteam und Telenotarzt-Zentrale 25
- kontinuierliche Übertragung von Vitalparametern (EKG, Pulsoxymetrie, Blutdruck, endtidales CO_2 - Kurven und Zahlenwerte -) (IntelliVue Information Center, Philips Healthcare); Die Monitor-Defibrillator-Einheit Heartstart MRx (Philips Healthcare) ist mit der Übertragungseinheit via Ethernet und Bluetooth verbunden. 25
- periodische Übertragung von Vitalwerten im einminütigen Intervall (Heartstart Telemedicine System, Philips Healthcare). 26
- Versand eines 12-Kanal-EKGs via Telemedizinsystem (Heartstart Telemedicine System, Philips Healthcare) oder via Mobilfax. 26
- Versand von Fotos, die mit einer Digitalkamera erzeugt werden. 26
- Übertragung von Auskultationsgeräuschen eines elektronischen Stethoskops (Littmann 3200, 3M). 26
- im Rettungswagen besteht zusätzlich die Möglichkeit einer Videoübertragung mit Hilfe einer fest verbauten Deckenkamera (SNC-RZ 50P, Sony Electronics Inc, USA). 26
Wenn das mobile System im Fahrzeug konnektiert wird, erfolgt automatisch die Umschaltung auf das stationäre System. 26
4.2.3 Einbindung von Medizinprodukten 26
Die Monitor-Defibrillator-Einheit Heartstart MRx der Fa. Philips Healthcare wird verwendet. Mit Hilfe einer Ethernetschnittstelle und Bluetooth ist diese mit der mobilen oder stationären Übertragungseinheit konnektiert und überträgt die Daten automatisiert an die Telenotarzt-Zentrale. Ein Unterbrechen dieser Übertragung ist durch Ausschalten der Übertragungseinheit jederzeit möglich. Es finden keinerlei Eingriffe in das eigentliche Medizinprodukt statt, sondern dieses ist lediglich zu

Anzeigezwecken von Vitalparametern durch dafür vom Hersteller vorgesehene Übertragungswege mit einer externen Stelle verbunden....26
Die Anzeige dieser Daten erfolgt ausschließlich über dafür von Philips zur Verfügung gestellte Software, die nach Medizinproduktegesetz (MPG) zertifiziert sind (IntelliVue Information Center, Heartstart Telemedicine System, beides Philips Healthcare)....26
Die Fa. Philips ist von Beginn an der Entwicklung des mobilen Netzwerkes zur Übertragung beteiligt. Daher konnten alle Anforderungen, die nach MPG-Zertifizierung an dieses Netzwerk bestehen, berücksichtigt werden. Nach Fertigstellung der Entwicklung wird die Fa. Philips das Netzwerk überprüfen und ein Zertifikat ausstellen, dass alle vom Hersteller geforderten Standards erreicht wurden. Da die Fertigstellung des Systems für Ende Oktober 2011 geplant ist, wird dieses Zertifikat der Ethikkommission des Universitätsklinikums Aachen nachgereicht....26
Ein elektronisches Stethoskop der Fa. 3M / Littmann wird mit Hilfe einer Bluetooth Schnittstelle ebenfalls mit den Übertragungseinheiten verbunden. Die Auskultationsgeräusche können in der Telenotarzt-Zentrale mit Hilfe eines zweiten elektronischen Stethoskops empfangen und gehört werden. Die dafür eingesetzte Software ist vom Hersteller explizit für diesen Zweck nach MPG zugelassen....26
Die Verwendung von Medizinprodukten und Nicht-Medizinprodukten in einem modularen System erfordert die Betrachtung und letztlich Beachtung des Medizinproduktegesetzes und der Medizinproduktebetreiberverordnung. Daher wurde Herr Prof. Fehn (Köln) als Gutachter beauftragt, die medizinprodukterelevanten, rechtlichen Fragestellungen vorab zu begutachten. Er kommt zu dem Ergebnis, dass der Einsatz des in diesem Prüfplan beschriebenen Systems gesetzeskonform ist, wenn die Geräte gemäß ihren Zweckbestimmungen eingesetzt werden. Die definierten Zweckbestimmungen der Geräte und des Systems sowie das Gutachten befinden sich im Anhang zu diesem Prüfplan....26
Alle anderen verwendeten Geräte (Digitalkamera, Deckenkamera) werden nicht zu Diagnosezwecken eingesetzt, sondern dienen als ergänzende Informationsquellen zur Sprachkommunikation. Jedoch erfolgt auch hier keinerlei Eingriff in die Produkte selber, sondern sie werden gemäß ihrer Bestimmung eingesetzt. Das System ist ein insgesamt modulares System, welches nicht den Gebrach aller Funktionalitäten gleichzeitig erfordert. Im Gegenteil werden im Sinne der Datensparsamkeit nur die Daten übertragen, die im konkreten Fall medizinisch erforderlich sind....27
4.2.4 Telekonsultation....27
Die Telekonsultation findet bei der überwiegenden Zahl der Einsätze zu Beginn zwischen den Rettungsassistenten vor Ort und einem Telenotarzt statt. Die Entscheidung, eine Telekonsultation durchzuführen obliegt dabei den Rettungsassistenten nach erfolgter Aufklärung des Patienten über diese Konsultation. In dieser Phase der Rettungsassistent-Arzt-Konsultation verfolgt die Telekonsultation das Ziel, ärztliche Expertise bereit zu stellen, in einer Einsatzphase, bei der (noch) kein Notarzt vor Ort ist. Fragen zu seltenen Erkrankungen und Medikamenteninteraktionen können beantwortet und Hilfestellung bei der Beurteilung des 12-Kanal-EKGs kann angeboten werden. Bei einigen Einsätzen führen Rettungsassistenten seit Jahren im Rahmen der sog. Notkompetenz ausgewählte ärztliche Maßnahmen wie i.v.-Zugang und Applikation ausgewählter Medikamente

selbstständig durch. Diese Maßnahmen können nun ärztlich begleitet und überwacht werden. Im Fall einer Komplikation, kann der Telenotarzt medizinische Anweisungen geben, die Folgeschäden verhindern können. Im Falle deutlich verlängerter Eintreffzeiten eines Notarztes können durch den Telenotarzt auch Maßnahmen an die Rettungsassistenten delegiert werden, die bisher im Regelfall von diesen nicht ohne Arzt vor Ort durchgeführt werden. Um den Patienten vor Schäden durch eine zu spät eingeleitete Therapie zu schützen, können durch den Telenotarzt, nach individueller ärztlicher Entscheidung im konkreten Einsatz, solche Medikationen delegiert werden. Beispielsweise ist die Delegation von Analgetikaapplikationen an das Pflegepersonal im Krankenhaus üblich, im Rettungsdienst jedoch die Ausnahme. Schwere Schmerzzustände oder andere akut bedrohliche Zustände erfordern jedoch eine möglichst zeitnahe, korrekte Medikation. In einem Pilotprojekt im Rettungsdienst Mittelhessen wurde bei insgesamt 172 Patienten Morphin algorithmusbasiert durch Rettungsassistenten zur Analgesie bei Extremitätentrauma appliziert. Dazu erfolgte eine telefonische Freigabe und Delegation durch einen Notarzt. In keinem dieser Fälle wurde eine bedrohliche Komplikation beobachtet und das Schmerzniveau konnte signifikant gesenkt werden.[26] Im Gegensatz zur telefonischen Delegation von Analgetika im Krankenhaus und zur rein telefonischen Delegation im Rahmen des Pilotprojektes in Mittelhessen, überwacht der Telenotarzt im Projekt TemRas den Patienten kontinuierlich weiter (EKG, Pulsoxymetrie, Blutdruck, Sprechverbindung mit Rettungsassistenten) bis dieser einem Arzt zugeführt ist (Notarzt trifft ein / Ankunft im Krankenhaus). Daher wird von einer zusätzlich erhöhten Sicherheit für den Patienten ausgegangen. Im Vorgängerprojekt Med-on-@ix wurden die rechtlichen Fragestellungen bezüglich der telemedizinischen Delegation ärztlicher Leistungen durch Gutachten von Herrn Prof. Katzenmeier und Herrn Prof. Fehn bereits umfassend beantwortet und der Ethikkommission des UKA zur Begutachtung vorgestellt (EK 141/09). Beide Gutachter kommen zu dem Ergebnis, dass die telemedizinische Delegation ärztlicher Leistungen grundsätzlich rechtskonform ist. Zudem betonen sie, dass dabei die Maßnahmen an Fachpersonal, das speziell für Notfallsituationen geschult ist, delegiert werden. Beide Gutachten befinden sich im Anhang dieses Prüfplans..........27

4.2 Datenquellen..........28
Folgende Datenquellen werden im Rahmen der wissenschaftlichen Evaluation verwendet:..........28
Notarzteinsatzprotokolle und Rettungsdienstprotokolle der teilnehmenden Rettungsdienste; aus diesen werden die Fälle identifiziert die detailliert ausgewertet werden (Einschlusskriterien)..........28
einsatzbezogene Zeiterfassung der Rettungsleitstelle zu Berechnung von Eintreff- und Versorgungszeiten..........28
Daten aus den Krankenhausinformationssystemen der Krankenhäuser, denen die eingeschlossenen Patienten zugeführt wurden: Diagnosen nach ICD 10, Zeitstempel diagnostischer und therapeutischer Verfahren (z.B. Computertomographie, Herzkatheteruntersuchung), Laborwerte, Schweregrad mit Hilfe fallbezogener Scoringsysteme (z.B. National Institutes of Health Stroke Scale beim Schlaganfall), Krankenhausverweildauer..........28
Dokumentation des Telenotarztes und empfangene Datenpakete in der Telenotarzt-Zentrale (z.B. EKG, Blutdruck…)..........28

Da es sich bei diesen Daten um klinische Routinedaten handelt, die nicht gesondert für diese Studie erhoben werden, ist von einer hohen Validität der Daten auszugehen. Zudem entsteht keinerlei Zusatzbelastung für das medizinische Personal und keine zusätzliche Erhebung und Speicherung von sensiblen, persönlichen Patientendaten....28
Nachdem ein Patient in die Studie eingeschlossen wurde, werden die erforderlichen Daten durch die Prüfärzte aus den nicht-anonymisierten Datenquellen (z.B. Krankenhausinformationssystem) heraus gelesen und in eine anonymisierte Datenbank übertragen. Die eigentlichen statistischen Auswertungen, an der auch nicht-ärztliches Personal wie Biostatistiker beteiligt sind, erfolgen dann vollständig anonymisiert. Somit erfolgt die Datenerhebung zwar prospektiv, die Auswertung hingegen hat jedoch eher den Charakter einer retrospektiven, anonymisierten Auswertung....28
Im Rahmen der Akzeptanzuntersuchungen werden anonymisierte Fragebögen sowohl von Rettungsassistenten, Notärzten und Telenotärzten ausgefüllt als auch anonymisierte Fragebögen an Patienten geschickt. Zudem erfolgt die Erhebung und anonymisierte Dokumentation qualitativen Feedbacks des Rettungsdienstpersonals im Rahmen von Gruppendiskussionen und Einsatzbesprechungen. Im Anhang sind die Fragebögen für Patienten und rettungsdienstliches Fachpersonal zu finden....28
Zur Erhebung von Strukturdaten der Rettungsdienste erfolgen Gespräche mit Verantwortlichen im jeweiligen Rettungsdienstbereich sowie fragebogenbasierte Abfragen bei diesen Personen. Auch hier wird auf Daten der Rettungsleitstelle zurückgegriffen. Dies sind jedoch keine patientenbezogenen Daten, sondern allgemeine statistische Daten wie z.B. „durchschnittliche Eintreffzeit“, „Notarztquote“, „Einsatzspektrum“....29
4.3 Risiko-Nutzen-Abwägung und Vorsichtsmaßnahmen....29
Wenn die Versorgung von Notfallpatienten durch einen Telenotarzt unterstützt wird, ist insgesamt von einer Erhöhung der Patientensicherheit auszugehen. Das in TemRas verwendete System wird lediglich als zusätzliche Unterstützung eingesetzt und ersetzt nicht bestehende Systeme. Es kommt in den meisten Fällen in einer Einsatzphase zur Anwendung, in der die Rettungsassistenten zum jetzigen Zeitpunkt, keinen ärztlichen Rat zur Verfügung haben. Mit dem Einsatz der Telekonsultation kann diese Versorgungslücke geschlossen werden und ärztlicher Rat steht umgehend zu Verfügung, was im Einzelfall sogar lebensrettend sein kann. Daher überwiegt aus heutiger Sicht klar der Nutzen die potentiellen Risiken. Die Abwägung zwischen Nutzen und Risiken, die Untersuchung der Risiken und die Festlegung der zu treffenden Vorsichtsmaßnahmen werden vor und während der Studie im Rahmen eines kontinuierlichen Risikomanagementprozesses durchgeführt....29
Folgende potentielle Risiken sind im Rahmen von Workshops unter Beteiligung aller Konsortialpartner identifiziert worden:....29
Ablenkung des Personals vor Ort durch die Telekonsultation....29
fehlgerichtete Kommunikation als Ursache für einen solchen Ablenkungseffekt....29
Fehlentscheidungen, bei partiellem oder totalem Ausfall von Komponenten oder Datenverbindungen 29
Ziel des Risikomanagementprozesses ist die Minimierung der Risiken des Systems für den Patienten indem drei wesentliche Eigenschaften des Netzwerks im Entwicklungsprozess sichergestellt werden: Sicherheit, Leistungsfähigkeit sowie Daten- und Systemschutz. Das Risikomanagement wird vom IMA

der RWTH Aachen verantwortet und unter Einbeziehung der anderen Partner durchgeführt. Um die Risiken minimal zu halten, werden im Rahmen des Risikomanagementprozesses Risiken identifiziert und bewertet, Gegenmaßnahmen festgelegt und der Prozess in einer Risikomanagementakte dokumentiert. 29

Der Risikomanagementprozess orientiert sich an der DIN EN ISO 14971 (Abbildung 1). In der Risikoanalyse werden Risiken identifiziert und ihre Schwere und soweit möglich Eintrittswahrscheinlichkeit geschätzt. In der Risikobewertung wird betrachtet, ob Maßnahmen durchgeführt werden sollten. Falls ja, werden in der Risikobeherrschung Maßnahmen festgelegt, evaluiert und durchgeführt. Falls dadurch neue Risiken entstehen, müssen diese wieder evaluiert werden. Zum Schluss muss das Gesamtrestrisiko bewertet werden. Alle Phasen des Risikomanagementprozesses werden durch Workshops unterstützt. Besonderes Augenmerk liegt auf einzelnen Komponentenausfällen, die für sich allein zum Ausfall des ganzen Systems führen könnten (Single-Points-of-Failure). 30

.... 30

Die Risikomanagementakte ist der zentrale Ort, an dem alle für das Risikomanagement relevanten Informationen gesammelt werden. Sie enthält Verweise auf die relevanten Entwicklungsdokumente (Anforderungen, Use-Cases, Standard Operating Procedures, BugTracker). Außerdem erfasst sie die Identifikation der Risiken und die dazu entschlossenen Gegenmaßnahmen. 30

Alle drei Monate finden Risikomanagement-Workshops statt, in denen im Sinn eines kontinuierlichen Verbesserungsprozesses der Stand der Umsetzung der Maßnahmen kontrolliert, die Maßnahmen auf ihre Effektivität überprüft und neue Risiken identifiziert werden. 30

Zur Reduktion der Risiken im Umgang der beteiligten Akteure mit dem System werden diese vor Beginn der praktischen Evaluationsphase intensiv im Rahmen standardisierter Schulungen auf den Einsatz des Systems ausgebildet. Hierbei liegt der Fokus neben der Einweisung auf die Übertragungstechnik besonders auf der möglichst einheitlichen und zielgerichteten Kommunikation. Diese soll eine strukturierte, effiziente und sichere Übermittlung von Gesprächsinhalten ermöglichen. Angelehnt wird diese Kommunikation an die Kommunikation im Funk für Behörden und Organisationen mit Sicherheitsaufgaben (BOS-Funk, Rettungsdienst, Feuerwehr, Polizei), die den Beteiligten aus ihrem beruflichen Alltag bestens bekannt ist. In den Schulungen werden Fallbeispiele durchgeführt, die zum Ziel haben, genau diese Kommunikation zu trainieren. Weiterhin werden rechtliche Themen wie Aufklärung des Patienten, rechtliches Verhältnis Rettungsassistent-Telenotarzt und haftungsrechtliche Fragen in das Schulungskonzept integriert. 30

In die Schulungen werden Fallbeispiele integriert, die zum Ziel haben, genau diese Kommunikation zu trainieren. Als weitere Sicherheitsebene wird der Telenotarzt durch softwarebasierte Checklisten unterstützt, um trotz räumlicher Distanz zum Patienten, keine wesentlichen diagnostischen und therapeutischen Schritte zu übersehen. Die Vorteile einer checklistenbasierten Arbeitsweise und die positiven Einflüsse auf die Patientensicherheit sind aus dem WHO-Projekt „Safe Surgery Saves Lifes“ bestens bekannt. Sowohl für elektive als auch notfallmäßige Operationen konnte ein verbessertes Outcome durch checklistenbasiertes Arbeiten im „Hochrisiko-Bereich OP-Saal“ nachgewiesen werden.[21-23] Ein solches Checklistenprinzip zur Sicherstellung des notwendigen Mindeststandards ist

auch auf die Telekonsultation übertragbar. In den SOP wird der Gebrauch dieser Checklisten für die Telenotärzte definiert. Beispielsweise darf die Delegation einer intravenösen Medikation erst dann erfolgen, wenn anhand der entsprechenden Checkliste alle notwendigen Informationen eingeholt (z.B. Allergien erfragt worden sind) und alle Sicherheitsmaßnahmen (z.B. Pulsoxymetrie, Blutdruckmessung, Rhythmus-EKG) ergriffen sind. Im Projekt Med-on-@ix wurden bereits positive Erfahrungen mit checklistenbasierter Telekonsultation beim Schlaganfall gemacht. Durch Einsatz einer „Stroke Checkliste“ in der Telenotarzt-Zentrale konnte die Menge der schlaganfallspezifischen Informationen für den Neurologen in der Klinik mehr als verdoppelt werden (p<0,0001) (ergänzende Daten siehe Anlage). Die Erstellung von SOP und Checklisten erfolgt durch die Projektmitarbeiter nach wissenschaftlichen Kriterien. Dabei muss ein Dokument und von allen beteiligten Prüfärzten geprüft und von der Projektleitung freigegeben werden. Für Überarbeitungen dieser Dokumente innerhalb der Projektlaufzeit gilt das gleiche Verfahren. 30

Zur Analyse der Kommunikationsstandards und zur Sicherstellung eines hochwertigen Kommunikationsniveaus, finden in der Telenotarzt-Zentrale regelmäßig Supervisionen durch Kommunikationswissenschaftler des IMA/ZLW & IFU der RWTH Aachen statt. Diese haben zum Ziel ein konstruktives Feedback an die Telenotärzte zu geben, um eine weitere Optimierung der Kommunikation in der Projektlaufzeit zu erreichen. Zudem ist die Telenotarzt-Zentrale immer mit zwei Telenotärzten besetzt. Dies garantiert neben der erhöhten Verfügbarkeit auch ein regelmäßiges inter-kollegiales Feedback. 31

Der Telenotarzt beurteilt die Güte und Zuverlässigkeit der Datenverbindungen bei jeder Telekonsultation. Sollte aufgrund geringer Übertragungsraten eine kontinuierliche Übertragung von Vitalparametern nicht möglich sein, so darf er laut SOP keine Delegationen durchführen, die eine solche Überwachung sinnvoll erscheinen lassen. Jedoch muss an dieser Stelle bedacht werden, dass der Patient selbst bei vollständigem Funktionsausfall des gesamten Telemedizinsystems immer noch (wie aktuell deutschlandweit) durch qualifizierte Rettungsassistenten betreut wird, die in lebensrettenden ärztlichen Maßnahmen geschult und trainiert sind. Um bei Totalausfall des Systems trotzdem zumindest eine Sprachkommunikation zwischen Rettungsteam und Telenotarzt zu ermöglichen, verfügt jeder Rettungswagen zusätzlich über ein Mobiltelefon (Backup-Handy) wie es auch in der DIN EN 1789 für Rettungswagen gefordert wird. Die Telenotarzt-Zentrale verfügt zudem über einen handelsüblichen Festnetzanschluss, der selbst bei Ausfall der Internetverbindung erreichbar ist. 31

Im Rahmen eines IT-Sicherheitskonzepts werden die in TemRas anfallenden Daten vor unberechtigtem Zugriff, unberechtigter Veränderung und Verlust geschützt. Es beginnt mit einer Übersicht über das System und definiert den Prozess zur kontinuierlichen Überarbeitung des Konzepts. Die in TemRas anfallenden Patientendaten und die Geräte, die die Daten verarbeiten werden identifiziert und Schutzmechanismen definiert. Ferner wird festgehalten, wie lange die Daten nach Einsatzende aufbewahrt werden, wie sie geschützt werden und wer Zugang zu Ihnen hat. 31

Vor diesem Hintergrund überwiegen die Nutzen-Effekte eines solchen Systems. Maßnahmen zur Reduzierung möglicher Risiken werden umfassend ergriffen. 32

4.4 Unerwünschte Ereignisse 32

Unerwünschte Ereignisse können die Faktoren Technik, Organisation oder Medizin betreffen. Im Falle eines solches unerwünschten Ereignisses, das im Rahmen einer Telekonsultation auftritt, erfolgt eine standardisierte Dokumentation. Sowohl die Telenotärzte als auch das Rettungsfachpersonal verfügen dafür über Vorlagen. Diese Dokumentationen werden unverzüglich analysiert, um mögliche Probleme und Gefahrenquellen frühzeitig zu erfassen und kurzfristige Gegenmaßnahmen innerhalb des Projektzeiraums ergreifen zu können....32
4.5 Interim Analysen....32
Monatlich werden umfassende Analysen der Dokumentationen der unerwünschten Ereignisse (falls angefallen) durchgeführt. Änderungen des Vorgehens und des Prüfplans erfolgen, falls notwendig, adaptiert an diese Ergebnisse....32
Eine Zwischenauswertung von medizinischen Zielparametern ist aufgrund der Komplexität der Daten nicht vorgesehen. Zudem gibt es aus dem Vorgängerprojekt Med-on-@ix keinerlei Hinweise und Daten auf eine mögliche negative Beeinflussung des Behandlungsablaufs. Sollten sich jedoch aus der Dokumentationen der unerwünschten Ereignisse Hinweise ergeben, dass eine Zwischenauswertung notwendig ist, so wird eine solche unverzüglich durchgeführt....32
4.6 Abbruch der Studie....32
4.6.1 Abbruch der Studie bei einem Probanden....32
Sollte es zu einem relevanten technischen Defekt im Einsatz kommen, so wird diese Telekonsultation abgebrochen. Es soll während einer Patientenversorgung kein Reparaturversuch am System unternommen werden. Nach dem Einsatz erfolgt dann die technische Diagnose und ggf. Reparaturmaßnahmen. Ein weiterer Grund wäre die sekundäre Ablehnung des Verfahrens durch Patienten, d.h. er zieht nach Beginn der Telekonsultation die Einwilligung in dieses Verfahren zurück. 32
4.6.2 Abbruch der gesamten Studie....33
Sollte es im Verlauf der Studie Anhaltspunkte dafür geben, dass es durch das Verfahren zu Schädigungen von Patienten kommt, so wird unverzüglich eine Interimsanalyse der bis dahin gewonnen Daten durchgeführt. Sollten sich solche Vermutungen bestätigen, würde dies zum Abbruch der Studie führen. Als mögliche Schädigungen seien genannt:....33
signifikant schlechtere Reanimationsergebnisse....33
signifikant verlängerte Versorgungszeiten bei lebensbedrohlichen, zeitkritischen Notfällen....33
signifikant häufigere Komplikationen bei telemedizinischer Begleitung von Medikamentenapplikation durch Rettungsfachpersonal....33
5. Biometrie....33
5.1 Biometrisches Design....33
Die Studie wird als Prä-post-Interventionsstudie durchgeführt. Mit Start der praktischen Evaluationsphase beginnt eine prospektive Datenkollektion vom 01.08.2012 und endet geplanter Weise am 31.07.2013. Die Daten des Prä-Interventionszeitraums werden folgendermaßen analysiert: Die Definition der auszuwertenden Daten erfolgt analog zur Post-Interventionsphase prospektiv. Jedoch erfolgt die eigentliche Auswertung retrospektiv. Es handelt sich bei den Daten nur um klinische

Routinedaten, nicht um gesondert für diese Studie erhobene Daten. Die statistische Auswertung erfolgt dann vollständig anonymisiert....33
5.2 Stichprobenplanung....33
5.3 Datenerfassung und Auswertung....34
Die Datenerfassung erfolgt wie oben beschrieben nur durch Prüfärzte des UKA. Die anonymisierte Datenauswertung erfolgt dann in Kooperation mit dem Institut für medizinische Statistik des UKA.....34
5.4 Statistische Methoden....34
Die primären und sekundären Outcomeparameter werden vor und nach der Intervention miteinander statistisch verglichen. Parametrische und nicht-parametrische statistische Verfahren kommen dabei zum Einsatz. Sollten sich mögliche Einflussfaktoren auf die Ergebnisse zeigen, so werden diese z.B. im Rahmen einer Varianzanalyse ausgewertet....34
6. Änderungen des Prüfplans....34
Sollten sich im Verlauf der Studie notwendige Änderungen im Prüfplan ergeben, so wird unverzüglich die Ethikkommission des UKA darüber informiert und die Änderungen im Studienregister publiziert....34
7. Ethische und rechtliche Belange....34
7.1 Rechtliche Grundlagen....34
RettG NRW: Alle dort geforderten Standards werden nicht berührt....34
Rechtsgutachten: In den beiden Rechtsgutachten von Prof. Fehn und im Gutachten von Prof. Katzenmeier[28] wird dargestellt, dass die Durchführung der hier geplanten Studie rechtskonform möglich ist. Dieer Inhalte der Rechtsgutachten werdenird den Rettungsassistenten in kompakter Form und den Telenotärzten in detaillierter Form im Rahmen der Schulungen vermittelt. Die vollständigen Rechtsgutachten können von jedem Beteiligten eingesehen werden....34
Berufsordnung für Ärzte: Die Berufsordnung für Ärzte wird nicht negativ berührt....34
Es werden umfassende Maßnahmen zur Sicherung der höchst schützenswerten Patientendaten, wie im Bundesdatenschutzgesetz gefordert, ergriffen....34
7.2 Votum der Ethikkommission....35
Da alle im Projekt in der Funktion als Telenotarzt eingesetzten Ärzte aus der Klinik für Anästhesiologie des Universitätsklinikum Aachen entstammen und die Telenotarzt-Zentrale auch durch diese Klinik betrieben wird, wird ein Ethikantrag bei der Ethikkommission des Universitätsklinikums Aachen eingereicht....35
Es erfolgt kein Beginn der praktischen Studienphase (Post-Interventionsphase) mit Telekonsultation ohne ein positives Ethikvotum....35
....35
7.3 3 Leiter der klinischen Studie....35
Dr. med. Jörg Brokmann (Ärztlicher Leiter Rettungsdienst Stadt Aachen, Oberarzt der Klinik für Anästhesiologie des UKA)....35
Univ.-Prof. Dr. med. Rolf Rossaint (Direktor der Klinik für Anästhesiologie des UKA)....35
7.4 4 weitere Prüfärzte....35
PD Dr. med. Stefan Beckers (Oberarzt Operative Intensivmedizin UKA)....35
Dr. med. Michael Czaplik (Anästhesiologie UKA)....35

Dr. med. Sebastian Bergrath (Anästhesiologie UKA)....35
Dr. med. Harold Fischermann (Anästhesiologie UKA)....35
Dr. med. Frederik Hirsch (Anästhesiologie UKA)....35
Daniel Wielpütz (Anästhesiologie UKA)....35
7.5 5 Archivierung und Datenschutz....35
Alle Patientendaten und einsatztaktischen Daten, die zu wissenschaftlichen Auswertungszwecken aufbewahrt werden müssen, werden sicher verschlossen im Universitätsklinikum Aachen in der Klinik für Anästhesiologie aufbewahrt. Dabei wird möglichst auf die Speicherung und Archivierung von identifizierbaren Merkmalen verzichtet sondern der pseudonymisierten und anonymsierten Form Vorrang gegeben. Nach der wissenschafltichen Auswertung, werden identifizierbare Daten vernichtet und lediglich die pseudonymisierten und anonymisierten Daten aufbewahrt....35
Alle regulären Patientendaten, die zu medizinischen Dokumentationszwecken archiviert werden, werden wie oben dargestellt, besonders gesichert auf Servern gespeichert....35
7.6 Versicherung der ärztlichen Tätigkeit als Telenotarzt....35
hier einfügen: Patientenversicherung etc....35
8. Publikation....36
Für die unter 2.2 aufgeführten Krankheitsbilder sollen die Ergebnisse in medizinischen Fachzeitschriften veröffentlicht werden. Dabei werden englischsprachige Journals angestrebt. Die Akzeptanzuntersuchungen sollen ebenfalls veröffentlicht werden. Hierbei sind sowohl deutschsprachige als auch englischsprachige Publikationen angestrebt....36
9. Zusammenfassung....36
Die telemedizinische Vernetzung zwischen medizinischem Personal und Spezialisten hat sich in vielen Bereichen der Medizin als vorteilhaft erwiesen. In der Notfall- und Akutmedizin kommen beispielsweise Telemedizinsysteme zwischen Krankenhäusern beim Schlaganfall zur Anwendung. Die Qualität der Patientenversorgung und das Outcome der Patienten konnten dadurch verbessert werden. In der präklinischen Notfallrettung kommt regelmäßig lediglich die Übertragung eines 12-Kanal-EKG an einen Kardiologen zum Einsatz. Für Patienten mit Myokardinfarkt konnten dadurch Versorgungszeiten verkürzt werden und letztlich das Outcome der Patienten verbessert werden. Andere telemedizinische Anwendungen kommen, abgesehen von zwei deutschen Pilotprojekten, nicht zum Einsatz. Der deutsche Rettungsdienst steht aktuell vor großen Herausforderungen. Speziell die Ressource „Notarzt“ ist in manchen Regionen nicht mehr flächendeckend verfügbar. Zudem hat die zunehmende Auslastung der Notarztstandorte durch stetig steigende Einsatzzahlen bereits zu merklich verlängerten Eintreffzeiten des Notarztes beim Patienten geführt. In aller Regel trifft ein Rettungswagen, besetzt mit qualifizierten Rettungsassistenten, bereits mehrere Minuten vor dem Notarzt beim Patienten ein, da das Standortnetz der Rettungswagen deutlich dichter ist als das der Notarztstandorte. In dieser Phase, in der (noch) kein Notarzt vor Ort ist, übernehmen die Rettungsassistenten eigenverantwortlich die medizinische Versorgung. Regelmäßig applizieren sie auch ausgewählte Medikamente und ergreifen ausgewählte invasive Maßnahmen vor Eintreffen des Notarztes....36

Um diese potentielle Versorgungslücke zwischen Eintreffen des Rettungswagen und Eintreffen des Notarztes zu minimieren, wird im Projekt TemRas (telemedizinisches Rettungsassistenzsystem) ein modulares, präklinisches Telemedizinsystem in einer einjährigen Evaluationsphase auf sechs Rettungswagen eingesetzt. Das System ermöglicht die Datenübertragung von Vitalparametern, 12-Kanal-EKGs, Auskultationsgeräuschen, Bildern und ggf. Videos an eine sog. Telenotarzt-Zentrale, die mit erfahrenen Notärzten (Telenotärzte) besetzt ist. Zu Diagnosezwecken verwendete Daten, werden nur über nach Medizinproduktegesetz zertifizierte Systeme, versendet. Im Rahmen dieser Telekonsultation wird eine Sprechverbindung zwischen den Rettungsassistenten und einem Telenotarzt aufgebaut. Der Telenotarzt kann das Team vor Ort in medizinischen und organisatorischen Fragen beraten. Zudem wird die Durchführung ärztlicher Maßnahmen zeitgleich überwacht. In ausgewählten Fällen kann über ein solches System auch die Delegation ärztlicher Maßnahmen erfolgen, wie beispielsweise die Delegation der Applikation von Analgetika vor Eintreffen eines Notarztes, um schwere Schmerzen frühzeitig zu lindern. Die Entscheidung ob eine Telekonsultation durchgeführt wird, obliegt grundsätzlich den Rettungsassistenten vor Ort. Wenn diese feststellen, dass ärztlicher Rat erforderlich ist, können sie die Telekonsultation durchführen...37

Die klinische, praktische Evaluation des Systems soll vom 01.08.2012 bis 31.07.2013 wochentags in der Zeit von 7.30 bis 16.30 Uhr im Rahmen einer Prä-post-Interventionsstudie erfolgen. Vor Einführung des Systems nehmen das Rettungsfachpersonal und die Telenotärzte an einem standardisierten Schulungskonzept teil. Es werden im Rahmen der praktischen Evaluationsphase (Post-Interventionsphase) prospektiv definierte Outcomeparameter aus klinischen Routinedaten analysiert. Es erfolgt anschließend der Vergleich mit einem einjährigen Zeitraum vor Schulung und Einführung des Systems. Die Daten dieses Prä-Interventionszeitraums werden retrospektiv und anonymisiert ausgewertet...37

10. Literatur...37

11. Anlagen..39

11.1 Übersicht Studien...39

11.2 Zweckbestimmung des Systems und der verwendeten Geräte..39

11.3 CE-Kennzeichnung von verwendeten Geräten..39

11.4 Rechtsgutachten TemRas Prof. Fehn 2011..39

11.5 Rechtsgutachten Med-on-@ix Prof. Katzenmeier 2009...39

11.6 Rechtsgutachten Med-on-@ix Prof. Fehn 2009...39

11.7 Ergebnisse Projekt Med-on-@ix (bisher nicht publizierte Ergebnisse)...39

11.8 Fragebögen für Patienten und Rettungdienstpersonal..39

Aachen, 04XX.0810.2011...39

11. Anlagen

1. Hintergrund

1.1 Hintergrund der klinischen Prüfung

In vielen Bereichen der Medizin hat sich die telemedizinische Vernetzung zwischen medizinischem Personal und fachspezifischen Spezialisten als vorteilhaft für die Versorgungsqualität erwiesen. In der Notfall- und Akutmedizin werden beispielsweise beim akuten Schlaganfall seit einigen Jahren national und international Telemedizinsysteme zwischen Krankenhäusern mit Stroke Unit und Krankenhäusern, die nicht über eine Stroke Unit verfügen, eingesetzt. Wissenschaftlich ist der Nutzen dieser Verfahren gut belegt.[1-5] In diesem Kontext hat sich die telemedizinische Vernetzung inklusive einer Videokonsultation in Echtzeit als vorteilhafter gegenüber der alleinigen Telefonkonsultation erwiesen.[1, 4] In der präklinischen Notfallmedizin sind die Vorteile einer prähospitalen Übertragung des 12-Kanal-EKGs an einen Kardiologen beim Myokardinfarkt nachgewiesen.[6-9] Es konnte sogar gezeigt werden, dass dadurch nicht nur Versorgungszeiten im positiven Sinne verändert werden, sondern dass sogar letztlich das Outcome der Patienten verbessert wurde.[10] Andere telemedizinische Anwendungen sind in der präklinischen Notfallmedizin sehr selten und wurden nur im Rahmen von Pilotprojekten realisiert.[11, 12] Von der American Heart Association, der weltweit größten Organisation zur Behandlung von Herz-Kreislauf-Erkrankungen, wird der Einsatz und die wissenschaftliche Evaluation von Telemedizinsystemen im Rettungsdienst empfohlen.[13-15] Im deutschen Projekt „Stroke Angel“ konnte gezeigt werden, dass durch die strukturierte Aufnahme von schlaganfallspezifischen Daten im Rettungsdienst mit Hilfe eines Tablet-Computers und deren automatisierte Weiterleitung an die aufnehmende Klinik, die klinischen Versorgungszeiten um fast die Hälfte reduziert wurden.[11] Mittlerweile ist „Stroke Angel“ der Übergang vom Pilotprojekt in den Regelbetrieb gelungen. Im Aachener Vorgänger-Projekt „Med-on-@ix“ (Ethikantrag EK 141/09) konnte gezeigt werden, dass eine mobile Telekonsultation zwischen dem Rettungsteam an der Einsatzstelle und einer sogenannten Telenotarzt-Zentrale sicher und ohne nachteilige Effekte für den Patienten machbar ist (siehe auch noch nicht publizierte Daten im Anhang).[16] Die Telekonsultation erfolgte in diesem Projekt fast ausschließlich zwischen dem Notarzt vor Ort und dem in einer sogenannten Telenotarzt-Zentrale befindlichen Telenotarzt. Die an der Versorgung beteiligten Rettungsassistenten konnten mit Hilfe einer Konferenzschaltung diese Konsultation mit verfolgen und sich bei Bedarf per Knopfdruck mit in das Gespräch einschalten. Insgesamt konnten keine negativen Einflüsse auf die Versorgungsqualität und an mehreren Stellen positive Einflüsse auf den Behandlungsablauf festgestellt werden. Das Projekt TemRas stellt eine technische und organisatorische Weiterentwicklung dar.

Das deutsche Rettungssystem setzt im Gegensatz zu anderen Ländern ein duales System aus qualifizierten Rettungsassistenten (zweijährige Berufsausbildung) und Notärzten ein. Während ungefähr die Hälfte aller Notfalleinsätze durch Rettungsassistenten alleine bewältigt wird, so wird in allen weiteren Notfällen zusätzlich ein Notarzt entsandt.[17] Diese sog. Notarztquote ist jedoch regional unterschiedlich, vor allem zwischen ländlichen und städtischen Bereichen. In der Stadt Aachen kam es im Jahr 2010 zu 22359 Einsätzen von Rettungswagen. In 7265 (32,5%) dieser Einsätze erfolgte

zusätzlich der Einsatz eines Notarztes, was deutlich unter dem Bundesdurchschnitt liegt (http://www.feuerwehr-aachen.de/index.php?id=24&L=0; aufgenommen am 14.06.2011). Die Notarztalarmierung geschieht dann primär, d.h. zeitgleich mit der Alarmierung des Rettungswagens, wenn die Rettungsleitstelle aus dem Notruf eine potentielle Lebensgefahr ableiten kann. Wird „nur" ein Rettungswagen zum Einsatzort entsandt, so evaluiert die Besatzung den Patientenzustand nach ihrem Eintreffen. Wenn die Situation bedrohlicher ist, als aus dem Notruf erkenntlich war oder spezielle Medikationen (z.B. Analgetika) notwendig sind, wird ein Notarzt nachträglich – nach Eintreffen des Rettungswagens – durch die Rettungsassistenten nachgefordert. In der Bundesrepublik ist das Standortnetz der Rettungswagen dichter als das der Notarztstandorte. Die landesrechtlichen Vorgaben der Rettungsgesetze definieren dabei die sogenannten Hilfsfristen als Planungsgröße für diese Standortnetze. Daher trifft in den meisten Fällen das Rettungswagenteam mehrere Minuten vor dem Notarzt beim Patienten ein und beginnt eigenverantwortlich mit der medizinischen Erstversorgung. Dieser Zeitversatz ist im ländlichen Raum ausgeprägter als in dicht besiedelten Gebieten. Aufgrund stetig steigender Einsatzzahlen und zunehmender Auslastung der Notarztstandorte ist jedoch eine direkte Verfügbarkeit eines Notarztes nicht in allen Fällen gegeben.[18] In Aachen beispielsweise stieg die Anzahl der Notarzteinsätze innerhalb von zehn Jahren von 5880 (2000) auf 7265 (2010) um 23% an, bei überwiegend unveränderter personeller Ausstattung. Das Zeitintervall von Alarmierung bis zum Eintreffen des Notarztes bei 95% aller Einsätze (sog. Sicherstellungszeit) hat sich in den letzten zehn Jahren bundesweit ebenfalls relevant verlängert (Deutscher Bundestag, Drucksache 16/2100 vom 28.06.2006). Von 1995 bis 2005 verlängerte sich dieses Zeitintervall von 18,6 auf 22,3 Minuten. Aus dieser Analyse wurden bereits die „Ausreißer" (d.h. 5%) im Sinne ungewöhnlich langer Eintreffzeiten ausgeschlossen. Im nordrheinwestfälischen Rettungsgesetz (RettG NRW) wird als Hilfsfrist für den Rettungswagen eine Zeit von 8 Minuten (städtischer Raum) bzw. 12 Minuten (ländlicher Raum) als Planungsgröße festgeschrieben. Alleine aus diesen Zahlen ist ersichtlich, dass das Zeitintervall zwischen dem Eintreffen des Rettungswagens und dem Eintreffen des Notarztes einen in vielen Fällen längeren Zeitraum darstellt.

Zudem sind einige Notarztstandorte aufgrund von Ärztemangel nicht immer kontinuierlich einsatzbereit, wie eine aktuelle Untersuchung aus Rheinland-Pfalz zeigt.[19] Auch wenn für die anderen Bundesländer diese exakten Daten nicht vorliegen, so muss jedoch von ähnlichen Problematiken ausgegangen werden. In diesen Fällen muss auf weiter entlegene Notarztstandorte oder tagsüber auf die Luftrettung zurück gegriffen werden, was wiederum mit verlängerten Eintreffzeiten des Notarztes beim Patienten einhergeht. Rettungsassistenten versorgen in diesen Fällen den Patienten medizinisch bis zum Eintreffen des Notarztes und führen sowohl ausgewählte invasive Maßnahmen wie peripher-venöse Zugänge und die Atemwegssicherung als auch intravenöse Medikationen eigenverantwortlich durch. Auch die Bundesärztekammer empfiehlt die Durchführung von Medikationen durch Rettungsassistenten, wenn gewisse Vorraussetzungen erfüllt sind (http://www.bundesaerztekammer.de/downloads/notfallkompetenz__medikamente.pdf; aufgenommen am 14.07.2011).

1.2 Notwendigkeit der klinischen Prüfung

Vor diesem Hintergrund soll wissenschaftlich überprüft werden, ob eine präklinische, multifunktionale Telekonsultation zwischen Rettungsassistenten und erfahrenen (Tele)Notärzten, Vorteile für die Patientenversorgung beinhaltet. Der Fokus liegt hierbei auf der Einsatzphase, in der die Rettungsassistenten auf sich alleine gestellt sind. Jedoch wird zusätzlich auch der Nutzen einer solchen Telekonsultation für das notärztliche Personal analysiert. Im Vorgängerprojekt Med-on-@ix konnte die technische und organisatorische Machbarkeit einer präklinischen, multifunktionalen Telekonsultation gezeigt werden.[16] Hierbei wurde die Konsultation jedoch hauptsächlich zwischen dem Notarzt vor Ort und einem räumlich getrennten Telenotarzt durchgeführt (Arzt-Arzt-Konsultation). Im Vergleich mit der Standardnotfallversorgung ohne Telekonsultation konnte gezeigt werden, dass Telekonsultation keinerlei Nachteile auf Einsatzzeiten und Versorgungsqualität hat. An einigen Stellen konnten positive Effekte gezeigt werden (siehe ergänzende Daten im Anhang). Der Vorteil einer Telekonsultation kommt besonders dann zum Tragen, wenn medizinisches Spezialwissen benötigt wird, über das das Personal vor Ort nicht verfügt. Im Projekt TemRas sind daher noch mehr positive Einflüsse auf die Patientenversorgung und eine erhöhte Patientensicherheit zu erwarten, da im Gegensatz zum Projekt Med-on-@ix die Telekonsultation zwischen nicht-ärztlichem Personal und einem Telenotarzt erfolgt. Während bei Med-on-@ix zumeist zwei gleich qualifizierte Ärzte telemedizinisch miteinander vernetzt waren, ist der Unterschied im medizinischen Wissen zwischen Rettungsassistenten und erfahrenen Notärzten jedoch deutlich größer. Daher ist häufiger eine sinnvolle Hilfestellung zu erwarten, wenn Rettungsassistenten in der Einsatzphase, in der (noch) kein Notarzt anwesend ist, einen Arzt konsultieren können.

In einer Simulationsstudie im Projekt Med-on-@ix wurde sowohl am Patientensimulator als auch mit Schauspielerpatienten der Vergleich zwischen Rettungsteams mit Notarzt vor Ort gegen Rettungsteams, die „nur telemedizinisch“ mit einem Notarzt vernetzt waren, durchgeführt. Dabei zeigte sich, dass die gemessene Versorgungsqualität beider Gruppen vergleichbar war. Rettungsassistenten waren unter Zuhilfenahme eines telemedizinisch verbundenen Notarztes in Bezug auf vorher definierte Qualitätskriterien nicht schlechter, als die Teams mit einem physisch anwesenden Notarzt vor Ort. Im Rahmen dieser Studie wurden jedoch bewusst Notfälle simuliert, die keine komplexen, invasiven Maßnahmen wie Narkoseeinleitung oder endotracheale Intubation erforderten. In zwei der überprüften Kriterien, waren die Teams in der Telemedizingruppe sogar signifikant besser als in der Kontrollgruppe (siehe auch noch nicht publizierte Daten im Anhang).

Nachdem die Arzt-Arzt-Konsultation im Rahmen einer klinischen Studie und die Rettungsassistent-Arzt-Konsultation im Rahmen einer Simulationsstudie überprüft wurden, folgt im Projekt TemRas nun die klinische Prüfung der Rettungsassistent-Arzt-Konsultation. Dabei erfolgt diese Verfahrensweise grundsätzlich rein additiv zur Standardnotfallversorgung, die weiterhin alle gesetzlichen Vorgaben erfüllt. In keinem Fall wird ein Notarzt durch diese Verfahrensweise eingespart, sondern es soll das Zeitintervall zwischen Eintreffen des Rettungswagens und Eintreffen des Notarztes telemedizinisch

durch erfahrene Notärzte begleitet werden. Ärztliche Maßnahmen werden dadurch ärztlich begleitet, mitverantwortet und letztlich überwacht.

1.3 Nutzen-Risiko-Abwägung

Der mögliche Nutzen einer umfassenden, präklinischen Telekonsultation zwischen Rettungsfachpersonal und einem Arzt ist bisher nicht ausreichend wissenschaftlich belegt. Es ist somit das Ziel dieser klinischen Studie, diese Frage zu klären. An dieser Stelle scheint die Definition des Begriffs „Telekonsultation" angebracht zu sein. Im Gegensatz zu einer rein telefonischen Konsultation (Telefonkonsultation) zwischen zwei Partnern werden bei einer Telekonsultation die Partner umfassend telemedizinisch miteinander vernetzt. Dabei werden zusätzlich zur Sprechverbindung auch Daten wie EKG, Sauerstoffsättigung oder Dokumente übertragen. Aus den Ergebnissen des Vorgängerprojektes Med-on-@ix ist bekannt, dass eine präklinische Telekonsultation zwischen zwei Ärzten die Versorgungszeiten nicht verlängert. Weiterhin kam es zu keinem Zeitpunkt zu einer dokumentierten Schädigung eines Patienten durch dieses Verfahren. In der Literatur gibt es klare Belege dafür, dass eine telemedizinische Vernetzung und zumindest eine kurzzeitige Telekonsultation zwischen nicht-ärztlichem Rettungspersonal und einem Kardiologen beim Myokardinfarkt positive Effekte auf die Patientenversorgung haben.[6,8,9] Eine Studie zeigte sogar eine Verbesserung des Outcome nach Myokardinfarkt.[10] Im deutschen Forschungsprojekt „Stroke Angel" kam es durch die telemedizinische Vernetzung von Rettungsassistenten und einer neurologischen Fachklinik zu positiven Effekten im Sinne verkürzter Therapieintervalle und einer erhöhten Lyserate beim Schlaganfall. Zwar kam es in diesem Projekt zu keiner bidirektionalen Sprechverbindung, jedoch mussten die Rettungsassistenten computerbasiert zusätzliche Informationen erheben und diese vor Eintreffen in der Zielklinik übertragen.[11]

Vor diesem Hintergrund gehen wir davon aus, dass das zusätzliche Risiko für den Patienten, das von einer Telekonsultation ausgeht, sehr gering ist. Im Gegenteil wird durch ärztliche Supervision in Echtzeit die Patientenversorgung vermutlich deutlich sicherer und Fehlentscheidungen, die gravierende Konsequenzen für den Patienten haben, können vermieden werden. Ärztlicher Rat steht mit Telekonsultation in einer Phase zur Verfügung, in der üblicherweise sonst keiner möglich ist. In standardisierten Schulungskonzepten werden sowohl die Rettungsassistenten als auch die Telenotärzte in einer zielgerichteten, effektiven Kommunikation geschult. Durch Vereinheitlichung und Standardisierung der Kommunikation soll möglichen Ablenkungseffekten entgegengewirkt werden. Erst nachdem das Personal diese Schulungen absolviert hat, kann es an dem Projekt teilnehmen. Zur Erarbeitung dieses Schulungskonzeptes erfolgt eine enge Kooperation mit Kommunikationswissenschaftlern des IMA/ZLW & IFU der RWTH Aachen und ein Austausch mit der Flugsicherungsakademie der Deutschen Flugsicherung in Langen. Die Verfahrensweise der Telekonsultation wird in Standard Operating Procedures (SOP) festgeschrieben, um eine möglichst einheitliche Prozessgestaltung zu gewährleisten. Diese SOP werden im Rahmen der Schulungen den Rettungsassistenten und den Telenotärzten vorgestellt und vermittelt. Ebenfalls werden rechtliche

Themen wie Patientenaufklärung, Verantwortlichkeiten, Zuständigkeiten und haftungsrechtliche Aspekte unterrichtet. Zusätzlich erfolgen durch Training in Fallbeispielen praktische Übungen, in denen diese Standards vor dem ersten Einsatz des Systems am Patienten eingeübt werden.

2. Studienziel

2.1 Studiendesign

Die Studie wird als prospektive Prä-post-Interventionsstudie durchgeführt. Vom 01.05.2012 bis 31.07.2012 erfolgen die oben erwähnten Schulungen des Personals. Ab dem 01.08.2012 soll eine zwölfmonatige klinisch, praktische Evaluation des Systems durchgeführt werden. In dieser eigentlichen Studienphase (Interventionsphase) entscheiden die Rettungsassistenten eigenständig, ob sie eine Telekonsultation durchführen möchten – analog zu der Entscheidung, ob ein Notarzt vor Ort gebraucht wird. Dadurch, dass der Rettungsassistent die Entscheidung zur (rein additiven) Telekonsultation trifft, wird einer möglichen Überforderung des Personals mit potentiell ablenkenden Effekten entgegengewirkt. Dies wird im Rahmen der vorherigen Schulungen dargestellt.

Es erfolgt der Vergleich mit einem einjährigen Zeitraum vor Schulung und Einführung des Systems. Dazu werden die gleichen, definierten Kriterien angewandt, wie bei der Evaluation des Interventionszeitraums. Die Analyse dieser Prä-Interventionsphase (Baselinedaten) erfolgt retrospektiv und letztlich anonymisiert nach Ablauf der Interventionsphase.

2.2 Zielparameter

Im Gegensatz zu Studien zur telemedizinischen Versorgung beim Schlaganfall oder Myokardinfarkt soll mit dieser Studie eine telemedizinische Versorgung nicht eines einzelnen Krankheitsbildes sondern aller Notfälle ermöglicht und analysiert werden. Daten zu folgenden Krankheitsbildern bzw. Symptomatiken sollen dabei nach vorher definierten, erkrankungsspezifischen Kriterien analysiert werden:

- akutes Koronarsyndrom
- Schlaganfall und intracerebrale Blutung
- akute Schmerzzustände
- hypertensiver Notfall
- Hypoglykämie
- Asthma bronchiale / chronisch obstruktive Lungenerkrankungen

Diese Notfälle stellen einen Großteil der rettungsdienstlich versorgten Patienten dar. Bei besonderen Ereignissen bzw. unerwünschten Ereignissen wird eine gesonderte, vorher definierte Dokumentation durchgeführt. Diese Ergebnisse fließen ebenfalls in die Auswertung mit ein.

Durch Vergleich dieser Zielgrößen vor und nach Einführung eines Telekonsultationssystems, soll der Einfluss der Einführung des Systems auf die Versorgungsqualität gemessen werden.

2.2.1 Primäre Zielgrößen

Die Darstellung der primären Outcomeparameter ist im Anhang in Tabelle 1 aufgeführt.

2.2.2 Sekundäre Zielgrößen

Die Darstellung der sekundären Outcomeparameter ist im Anhang in Tabelle 1 aufgeführt.

2.2.3 Weitere Studienparameter

Bei allen anderen Krankheitsbildern erfolgt sowohl die Analyse der Eintreff- und Versorgungszeiten als auch die Analyse von Komplikationen und besonderen Ereignissen. Zudem wird untersucht, wie häufig Patienten nach Untersuchung und Behandlung durch den Rettungsdienst zu Hause bleiben und nicht einem Krankenhaus zugeführt werden. Prä- und Postinterventionszeitraum werden dabei verglichen.
Da die an der Studie teilnehmenden Rettungsdienstbereiche eine sehr heterogene Gruppe darstellen (verschiedene Betreiber, Qualifikation des Personals unterschiedlich, städtisch vs. ländlich…) werden diverse Baselinedaten erhoben, um solche Unterschiede messbar zu machen. Da mit der Einführung und Schulung von Telekonsultation ein Bündel an Interventionen erfolgt, muss der Einfluss auf verschiedene Qualitätsindikatoren untersucht werden. Dazu zählen:

- Dispositionsqualität der Leitstelle, gemessen an dem Anteil korrekt disponierter RTW- und Notarzteinsätze, Vergleich Prä- und Postinterventionszeitraum
- Einsatzspektrum der RTW (Notarztquote, Anteil von Tracerdiagnosen), Vergleich Prä- und Postinterventionszeitraum
- Qualifikation und demographische Daten des Personals, Anteil ehrenamtlicher Mitarbeiter und Teilzeitkräfte, Vergleich Prä- und Postinterventionszeitraum
- Analyse des bisherigen Fortbildungsangebots in den Rettungsdienstbereichen im Prä- und Postinterventionszeitraum
- Anzahl der RTW und notarztbesetzten Rettungsmittel im Prä- und Postinterventionszeitraum
- Struktur der Voranmeldung im Krankenhaus: Mobiltelefon, 12-Kanal-EKG-Fax, Leitstelle, ltd. Notarzt; Vergleich Prä- und Postinterventionszeitraum
- Dokumentationsqualität der rettungsdienstlichen Dokumentation
- weitere Qualitätsindikatoren: geregeltes Notkompetenzsystem vorhanden?, Feedbacksystem an Mitarbeiter vorhanden?, Algorithmen / SOP implementiert?

Zusätzlich zu diesen Fragestellungen werden begleitend Akzeptanzuntersuchungen durchgeführt. Hierbei sind die Zielgruppen zum einen das notfallmedizinische Personal und zum anderen die durch den Rettungsdienst versorgten Patienten.
Die Akzeptanz des beteiligten Rettungsdienstpersonals wird im Projektverlauf in drei Phasen erhoben. In Anlehnung an bewährte Methoden der empirischen Sozialforschung wird die Akzeptanz unterteilt in Einstellungs-, Handlungs- und Nutzungsakzeptanz.[20] Die Rettungsdienstmitarbeiter werden sowohl

vor Beginn der Evaluationsphase (nach einer ersten Informationsveranstaltung), als auch während den vorbereitenden Schulungen (nach ersten Fallbeispielen und praktischen Trainings) sowie im Verlauf der eigentlichen klinischen Evaluation (während des Betriebs des Systems) hinsichtlich relevanter Akzeptanzfaktoren befragt. Mit der frühzeitigen Einbeziehung der Nutzer werden nicht nur Ängste und Hemmungen von Seiten der Rettungsdienste erhoben und ggf. minimiert, sondern das frühe Feedback erlaubt auch ggf. Anpassungen am System bzw. den organisatorischen Abläufen, die den Erfolg des Einsatzes stärken.

Die Befragungen des Rettungsdienstpersonals werden mit Hilfe von standardisierten Fragebögen und moderierten Gruppendiskussionen durchgeführt, die anonymisiert ausgewertet werden. Persönliche Daten, die eine eindeutige Identifikation ermöglichen, sind darin nicht enthalten. Während der klinischen Evaluation werden zudem qualitatives Feedback im Rahmen von Einsatzbesprechungen und Teamsitzungen dokumentiert und zur Qualitätssicherung genutzt. Auch hierbei erfolgt keine Dokumentation von persönlichen Daten.

Die Befragung der Patienten erfolgt rein fragebogenbasiert. Es werden sowohl Patienten im Präinterventionszeitraum (hierbei 01.12.2011 bis 30.04.2012) als auch im Postinterventionszeitraum angeschrieben und diese Daten miteinander verglichen. Die Entwicklung dieses Fragebogens erfolgt – wie im Projekt Med-on-@ix – unter Zusammenarbeit mit dem Institut für medizinische Psychologie des Universitätsklinikums Aachen und dem IMA/ZLW & IfU der RWTH. Der Patient wird dazu persönlich angeschrieben, was den Zugriff auf Patientendaten notwendig macht. Dieser Zugriff erfolgt ausschließlich durch die Prüfärzte. In einem kurzen Anschreiben wird auf die Freiwilligkeit zur Teilnahme an der Befragung deutlich hingewiesen. Dem Fragebogen liegt ein frankierter Rückumschlag bei, so dass keine Kosten für den Patienten entstehen. Der Zeitaufwand für das Ausfüllen des Fragebogens beträgt 10 bis 15 Minuten. Im Fragebogen befinden sich keinerlei Angaben, die eine Identifikation des Patienten ermöglichen. Lediglich eine Zuordnung zur Prä- und Postinterventionsgruppe kann erfolgen. Die statistische Auswertung erfolgt anonymisiert. Von dieser Befragung ausgeschlossen werden minderjährige Patienten, psychiatrische Notfallpatienten, demenzerkrankte Patienten und Patienten, die während des Einsatzes eine eingeschränkte Bewusstseinslage (Glasgow Coma Scale < 15) aufwiesen.

3. Probandenrekrutierung

3.1 Anzahl der Probanden und Dauer der Studie

Die praktische Anwendungs- und Studienphase mit Durchführung von Telekonsultationen wird über einen Zeitraum von 12 Monaten vom 01.08.2012 bis 31.07.2012 durchgeführt. In dieser Zeit wird die Telenotarzt-Zentrale montags bis freitags von 7.30 bis 16.30 Uhr mit zwei Telenotärzten von der Klinik für Anästhesiologie des Universitätsklinikum Aachen besetzt. Ein 24-Stunden-Betrieb ist aufgrund der Fördersumme nicht möglich. Die Schulungen der Rettungsdienstmitarbeiter erfolgen bereits in den drei Monaten vor Beginn der praktischen Studienphase.

Da insgesamt sechs Rettungswagen aus fünf unterschiedlichen Rettungsdienstbereichen den Telekonsultationsdienst in Anspruch nehmen können und die Einsatzzahlen variabel sind, ist an diesem Punkt lediglich eine Abschätzung der Fallzahl möglich. Dabei dienen folgende Zahlen als Berechnungsgrundlage:

Anzahl der Rettungswageneinsätze in den Wachbezirken (7.30 – 16.30 Uhr):

Aachen – Hauptwache:	4, davon 1 mit Notarzt
Aachen – Wache Süd:	2, davon 1 mit Notarzt
Köln – Wache Chorweiler:	4, davon 1 mit Notarzt
Kreis Düren – Wache Nideggen:	3, davon 1 mit Notarzt
Kreis Heinsberg - Wache Übach-Palenberg:	2, davon 1 mit Notarzt
Kreis Euskirchen – Wache Tondorf:	1, davon 0,5 mit Notarzt

Somit ergibt sich eine geschätzte Gesamtanzahl von 16 Rettungswageneinsätzen pro Studientag im Zeitraum von 7.30 bis 16.30 Uhr. Bei ca. 5,5 dieser Einsätze erfolgt der parallele Einsatz eines Notarztes. Nach lokaler Expertenmeinung ist daher von 5 bis 6 Telekonsultationen pro Tag auszugehen.

Bei 250 Arbeitstagen in der Post-Interventions-Phase ergibt sich somit eine Anzahl von 1250 bis 1500 Konsultationen.

3.2 Auswahl der Probanden

3.2.1 Einschlusskriterien

Bei notfallmedizinischen Studien werden häufig Patienten in Studien eingeschlossen, die zu zum Zeitpunkt der Behandlung nicht geschäftsfähig sind aufgrund einer Bewusstseinsstörung. Da in der Notfallmedizin häufig ein enormer Zeitdruck vorherrscht ist die Aufklärung über medizinische Maßnahmen stets an die zeitliche Dringlichkeit einer Maßnahme anzupassen. Dies wird so auch in

den Rechtsgutachten aus dem Projekt Med-on-@ix von Herrn Prof. Katzenmeier (S. 47ff und S. 75ff) und Herrn Prof. Fehn (S. 5ff) dargestellt und bestätigt.

Insgesamt müssen drei verschiedene Aufklärungen und Einwilligungen des Patienten dabei differenziert werden:

1. Aufklärung und Einwilligung in die Zuschaltung des Telenotarztes inklusive Datenübertragung (in Bezug auf § 203 Abs. 1 Nr. 1 und Abs. 2 Nr. 1 StGB)
2. Aufklärung und Einwilligung in invasive, medizinische Maßnahmen (in Bezug auf § 228 StGB)
3. Aufklärung und Einwilligung in die anonymisierte, wissenschaftliche, statistische Auswertung

zu 1.:

siehe dazu auch S. 5-8 sowie S. 47-49 Gutachten Prof. Fehn 2008

siehe dazu auch S. 47-51 und S. 75-76 Gutachten Prof. Katzenmeier 2009

Beide Gutachter kommen zu dem Ergebnis, dass zunächst die Aufklärung des Patienten über die Zuschaltung eines Telenotarztes erforderlich ist, wenn der Patient geschäftsfähig ist und zeitgleich keine höchste Dringlichkeit zur medizinischen Behandlung vorliegt. Der Patient muss nach der Aufklärung in diese telemedizinische Kommunikation einwilligen. Beide Gutachter beschränken jedoch diese Aufklärungspflicht auf die hier genannten Umstände und weisen darauf hin, dass viele Notfallpatienten nicht geschäftsfähig sind aufgrund von Schock, Schmerzzuständen oder Bewusstseinsstörungen. In solchen Fällen muss von der mutmaßlichen Einwilligung des Patienten ausgegangen werden. Beide Gutachter kommen ebenfalls zu dem Ergebnis, dass Aufklärung und Einwilligung auch bei geschäftfähigen Patienten entfallen können, wenn höchste Eile zur Lebensrettung oder Abwendung schwerer gesundheitlicher Schäden geboten ist. Diese Situation tritt in der Notfallrettung ebenfalls regelmäßig auf. Herr Prof. Fehn betont zudem, dass zusätzlich zur Aufklärung über die telemedizinische Zuschaltung des Telenotarztes meistens eine Aufklärung über invasive Maßnahmen (in Bezug auf §228 StGB) zu erfolgen hat. Er stellt heraus, dass bei den in der Notfallmedizin begrenzten zeitlichen Ressourcen speziell dieser Aufklärung über invasive Maßnahmen besondere Aufmerksamkeit zu gelten hat (siehe S. 8, Fehn 2008).

Eine schriftliche Aufklärung mit Hilfe eines Patientenaufklärungsbogens erfolgt bisher im deutschen Rettungsdienst grundsätzlich nicht. Lediglich bei einem Verzicht seitens des Patienten auf eine weitere Behandlung bzw. Transport in ein Krankenhaus erfolgt eine schriftliche Aufklärung, zumeist mit Unterschrift des Patienten und Zeugen. Allerdings ist der Patient niemals verpflichtet bei Verzicht auf eine notärztliche Behandlung eine solche Aufklärung zu unterschreiben. Sehr wohl aber müssen der Notarzt und der Rettungsassistent diesen Verzicht und die erfolgte Aufklärung über daraus resultierende Risiken möglichst exakt dokumentieren. Auf Notarzteinsatzprotokollen und Rettungsdienstprotokollen nach Empfehlungen der DIVI (Deutsche Interdisziplinäre Vereinigung für Intensiv- und Notfallmedizin) befindet sich für diesen Fall ein spezieller Dokumentationsbogen auf der Rückseite der Protokolle.

Vor diesem Hintergrund ist es zu bezweifeln, ob in einer medizinischen Notfallsituation ausreichend Zeit für den Patienten besteht, einen Aufklärungstext kritisch zu lesen, zu verstehen und dann nach einer Bedenkzeit sein schriftliches Einverständnis zu geben. Herr Prof. Fehn empfiehlt in seinem

Gutachten eine Standardaufklärung und ein Standarddokumentationsmodul. Daher wird im Projekt TemRas folgendermaßen verfahren:

Es erfolgt immer eine mündliche Aufklärung über die telemedizinische Konsultation des Telenotarztes. Diese Aufklärung erfolgt stets durch einen Rettungsassistenten und nicht durch Rettungsdienstpersonal geringerer Qualifikationsstufe (z.B. Rettungssanitäter). Sie erfolgt möglichst standardisiert mit Anpassungen an den konkreten Einsatzfall. Das Thema Patientenaufklärung wird in das Schulungskonzept für Rettungsassistenten und Telenotärzte integriert. Falls der Patient in die telemedizinische Konsultation einwilligt, kann diese durch die Rettungswagenbesatzung gestartet werden. Falls der Patient die telemedizinische Konsultation ablehnt, so ist diese Entscheidung bindend und die Rettungsassistenten verfahren so wie im Regelrettungsdienst üblich und entscheiden selbstständig ohne ärztliche Unterstützung. Bei Bedarf muss dann ein Notarzt hinzugezogen werden. Es erfolgt immer eine schriftliche Dokumentation über die Aufklärung und die Einwilligung bzw. Ablehnung auf dem Rettungsdienstprotokoll nach Empfehlung der DIVI. Ausnahmen von dieser Verfahrensweise sind nur möglich bei Patienten, die aufgrund ihrer Erkrankung oder Verletzung nicht mehr geschäftsfähig sind (z.B. im Rahmen einer Bewusstseinsstörung) oder wenn höchste Eile zur Lebensrettung geboten ist. In solchen Fällen muss der Rettungsassistent diese Ausnahme ebenfalls schriftlich exakt dokumentieren.

Nach Einwilligung bzw. bei mutmaßlicher Einwilligung initiiert die Rettungswagenbesatzung die Telekonsultation. Der Telenotarzt erfragt zu Beginn des Gesprächs, ob die Einwilligung des Patienten vorliegt und dokumentiert diese Einwilligung in einem standardisierten Dokumentationsmodul in der Telenotarztzentrale. Für den Fall, dass ein Patient im Rahmen einer Telekonsultation medizinische Maßnahmen ablehnt oder sogar den Transport ins Krankenhaus verweigert, erfolgt zusätzlich zur mündlichen Aufklärung eine schriftliche Aufklärung über daraus resultierende Risiken und eine Unterschrift des Patienten (und ggf. Zeugen) wird angestrebt. Diese schriftliche Dokumentation erfolgt auf dem dafür vorgesehen Bogen (Rückseite) des Notarzteinsatzprotokolls bzw. des Rettungsdienstprotokolls (gemäß Empfehlung der DIVI).

zu 2.:

Die Aufklärung über medizinisch-invasive Maßnahmen erfolgt – wie im Rettungs- und Notarztdienst üblich – immer mündlich und angepasst an die vorliegende Situation. Falls im Rahmen einer Telekonsultation invasive Maßnahmen an Rettungsassistenten delegiert werden, so muss der Rettungsassistent den Patienten zuvor über diese Maßnahme aufklären und dessen Einwilligung einholen. Der Telenotarzt hat zu kontrollieren, ob diese Aufklärung und Einwilligung stattgefunden hat, bevor die Maßnahme durchgeführt wird. Es erfolgt eine schriftliche Dokumentation durch die Rettungsassistenten auf dem DIVI-Rettungsdienstprotokoll und eine schriftlich-elektronische Dokumentation durch den Telenotarzt im dafür vorgesehen Dokumentationsmodul der Telenotarztzentrale. Ausnahmen von dieser Regelung betreffen analog zu Punkt 1 nicht geschäftsfähige Patienten (z.B. bei Bewusstlosigkeit) oder wenn höchste Eile zur Lebensrettung geboten ist (z.B. arterielle Gefäßverletzung). Eine solche Ausnahme wird im Anschluss ebenfalls exakt vom Rettungsassistent als auch vom Telenotarzt dokumentiert.

zu 3.:

Zunächst einmal gilt, dass ein Patient in wissenschaftliche Auswertungen seiner Daten einwilligen muss. Dies wird so auch im Rechtsgutachten von Herrn Prof. Katzenmeier bestätigt (S. 173-174, Katzenmeier 2009). Jedoch stellt der Gutachter im weiteren Verlauf dar, dass nach § 13 Abs. 2 Nr. 8 BDSG die Erhebung sensitiver personenbezogener Daten für Forschungszwecke zulässig ist, „soweit dies zur Durchführung wissenschaftlicher Forschung erforderlich ist, das wissenschaftliche Interesse an der Durchführung des Forschungsvorhabens das Interesse des Betroffenen an dem Ausschluss der Erhebung erheblich überwiegt und der Zweck der Forschung auf andere Weise nicht oder nur mit unverhältnismäßigem Aufwand erreicht werden kann.“ Für das Projekt Med-on-@ix wurde das Zutreffen dieser Regelung durch den Gutachter bestätigt. Das Folgeprojekt TemRas erfüllt die genannten Voraussetzungen für das Zutreffen dieser Regelung umso mehr, da nun durch Ausweitung des Systems auf fünf Rettungsdienstbereiche nicht nur regionale sondern durchaus repräsentative Daten (fünf Rettungsdienstbereiche mit unterschiedlichen regionalen Strukturen) erhoben werden. Das Gesamtinteresse dieser Daten für das Gesundheitssystems in Deutschland ist somit deutlich höher einzustufen als im Projekt Med-on@ix. Weiterhin gilt es zu bedenken, dass die Nutzung der patientenbezogenen Daten zu Forschungszwecken rein anonymisiert erfolgt. Es werden keinerlei zusätzliche patientenbezogene Daten erhoben, die ohne die geplante wissenschaftliche Evaluation des Systems (z.B. in einem Regelbetrieb) nicht erhoben würden. Einzige Ausnahme stellen hier die auf freiwilliger Basis erfolgenden fragebogenbasierten Akzeptanzuntersuchungen dar. Zudem sind anonymisierte statistische Auswertungen zu Qualitätsmanagementzwecken gesetzlich vorgeschrieben (z.B. Analyse der Eintreff- und Versorgungszeiten zur Erstellung eines Bedarfsplan nach §8 RettG NRW) und von der Bundesärztekammer (siehe http://baek.de/downloads/Aerztlicher_Leiter_Rettungsdienst_Empfehlung_BAeK_06_11_23_.pdf) empfohlen.

Jedoch soll der Patient im Projekt TemRas trotz dieser oben genannten Möglichkeit so gut wie möglich über das Forschungsprojekt informiert werden und mögliche Ängste bezüglich einer Datenauswertung abgebaut werden. Daher wird bei TemRas folgendermaßen verfahren:

Der Patient wird über das Forschungsprojekt und die anonymisierte Auswertungen seiner Daten durch die Rettungswagenbesatzung aufgeklärt, wenn die Einsatzumstände dies ermögliche (keine akute Lebensgefahr, Patient geschäftsfähig). Zusätzlich erhält jeder Patient – wie in den Rechtsgutachten empfohlen – eine standardisierte Patienteninformation (siehe Anlage). Somit kann sich jeder Patient nach seiner Genesung bzw. wenn er wieder voll geschäftsfähig ist umfassender über das Forschungsprojekt informieren und über die genannten Kontaktmöglichkeiten (Telefon, e-Mail, Postweg) offene Fragen klären. In einem persönlichen Arzt-Patienten-Gespräch können die genannte „anonymisierte Datenauswertung“ genauer erklärt werden und mögliche Ängste abgebaut werden. Ein solches Gespräch erfolgt immer durch einen im Forschungsprojekt involvierten Arzt. Eine Anonymisierung und anschließende Auswertung der Daten von Patienten ist laut Rechtsgutachten von Prof. Katzenmeier 2009 durch die Regelung nach § 13 Abs. 2 Nr. 8 BDSG somit rechtskonform.

Einschlusskriterien: Der Rettungsassistent entscheidet anhand des medizinisches Schweregrades und der Komplexität des Einsatzgeschehens, ob ärztlicher Rat und Expertise erforderlich sind. Ist dies der Fall, kann er unabhängig vom konkreten Krankheitsbild, Geschlecht oder Alter des Patienten die Telekonsultation durchführen. Der Patient muss nach mündlicher Aufklärung in die Telekonsultation einwilligen, bevor diese initiiert wird. Bei minderjährigen Patienten müssen dazu die Sorgeberechtigten einwilligen. Es erfolgt eine mündliche Aufklärung über die anonymisierte wissenschaftliche Datenauswertung. Eine schriftliche Aufklärung, inklusive Bedenkzeit in einer (potentiell) lebensbedrohlichen Notfallsituation ist nicht praktikabel und sinnvoll und könnte sogar lebensrettende Maßnahmen verzögern bzw. Ängste beim Patienten auslösen.. Im Rechtsgutachten von Prof. Katzenmeier wird zudem bestätigt, dass anonymisierte Datenauswertungen in der notfallmedizinischen Forschung auch ohne gezielte Aufklärung des Patienten erfolgen können, da sie von übergeordnetem Interesse im Sinne der Volksgesundheit sind (nach § 13 Abs. 2 Nr. 8 BDSG, s.o.). Dies ist Analogie zu einer retrospektiven, anonymisierten Auswertung zu sehen, bei der zumeist auch kein Einvewrständnis des Patienten eingeholt wird.

Beispiele aus der notfallmedizinischen Forschung: Eine Aufklärung von Patienten über die Teilnahme an Studien zur Evaluation des WHO-Projektes „Safe Surgery Saves Lifes“ erfolgte nicht, da die Auswertung letztlich anonymisiert erfolgte und man nicht davon ausging, dass dieses Verfahren ein Risiko für den Patienten darstellt.[21-23] Es wurde – ähnlich wie bei TemRas - ein Bündel von Interventionen eingeführt, um die Qualität der Patientenversorgung und die Patientensicherheit zu verbessern.

Selbst in einer Studie zur Medikamentenapplikation beim Herz-Kreislauf-Stillstand im randomisierten Studiendesign (keine vs. Standardmedikation) erfolgte – weil nicht möglich – keine Aufklärung, obwohl die mögliche Tragweite unter Umständen erheblich war.[24] Ebenso wurden – weil auch hier nicht möglich – die Patienten in einer Studie zur Thrombolyse bei Reanimation nicht aufgeklärt. Randomisiert erhielten sie entweder Placebo oder ein Thrombolytikum.[25]

Im Projekt TemRas gestaltet sich diese Situation deutlich weniger schwierig. Nach Schulung und Einweisung wird ein rein additives System im Rettungsdienst eingeführt, das einen zusätzlichen Dienst ergänzend zur Standardversorgung anbietet. Dieser Dienst stellt höchstwahrscheinlich einen deutlichen Mehrwert für den Patienten dar und erhöht höchstwahrscheinlich dessen Sicherheit. Nach Einführung steht dieses System jedem Patienten im Studienzeitraum zur Verfügung und keinem Patienten wird, z.B. durch eine Randomisierung, die Telekonsultation verweigert. Über die Datenübertragung und die Einschaltung des Telenotarztes muss ein geschäftsfähiger Patient aufgeklärt werden und in diese einwilligen, bevor diese gestartet wird Bei nicht geschäftsfähigen Patienten wird von einer mutmaßlichen Einwilligung in die Datenübertragung im Rahmen einer Geschäftsführung ohne Auftrag ausgegangen. Es wird, wie oben dargestellt, eine mündliche Aufklärung des Patienten über die anonymisierte Auswertung patientenbezogener Daten angestrebt, soweit dies aufgrund der Einsatzsituation möglich ist. Die Aufklärung des Patienten (bzw. die Unmöglichkeit einer solchen Aufklärung) werden schriftlich dokumentiert.

3.2.2 Ausschlusskriterien

Aus den Erfahrungen des Projektes Med-on-@ix ist eine Telekonsultation, die zeitgleich mit der Anamneseerhebung, Untersuchung und Behandlung des Patienten durchgeführt wird, bei der Gruppe der psychiatrischen Notfallpatienten in einigen wenigen Fällen nicht zuträglich. Zwar liegen dafür keine belastbaren wissenschaftlichen Daten vor, jedoch war dies der Eindruck des notärztlichen Personals im Projekt. Bei vielen dieser psychiatrischen Notfälle wurde daher im Projekt Med-on-@ix keine zeitgleiche Telekonsultation durchgeführt. Daher werden im Projekt TemRas psychiatrische Notfallpatienten primär vom Verfahren der Telekonsultation ausgeschlossen. Sollte ein Rettungsassistent jedoch in einem solchen Fall ärztlichen Rat benötigen, so kann er im Rahmen einer Einzelfallentscheidung, die Sprech- und Datenverbindung zum Telenotarzt aufbauen. Dies wäre beispielsweise möglich und sinnvoll, wenn Rettungsassistent und Patient räumlich getrennt voneinander sind oder wenn der Telenotarzt eine Voranmeldung in einer psychiatrischen Fachklinik durchführen soll.

4. Studiendurchführung

4.1 Methoden

Die Telekonsultation erfolgt zwischen einem Rettungswagenteam und einer Telenotarzt-Zentrale. Nach Eintreffen eines Notarztes kann dieser bei Bedarf zusätzlich in die Telekonsultation eingebunden werden oder die Telekonsultation wird nach Übergabe an den Notarzt vor Ort beendet.

4.1.1 Telenotarzt-Zentrale

Die Telenotarzt-Zentrale wird mit erfahrenen Notärzten der Klinik für Anästhesiologie des Universitätsklinikums Aachens besetzt. Dazu werden ausschließlich Ärzte mit der Zusatzweiterbildung Notfallmedizin und mindestens Facharztstandard eingesetzt. Zudem verfügen die Ärzte über spezielle notfallmedizinische Zusatzqualifikationen in Reanimation, Herz-Kreislauf-Notfällen und Traumaversorgung (Advanced Life Support Course, Pre-Hospital Trauma Life Support). Ihr Einsatz in der Funktion als Telenotarzt erfolgt erst nach der oben erläuterten standardisierten Schulung.

Die Telenotarzt-Zentrale wird räumlich bei der Berufsfeuerwehr Aachen eingerichtet. An zwei gleichartigen Bildschirmarbeitsplätzen können zwei Telenotärzte parallel Einsätze bearbeiten. Die Anzeige von Vitalparametern (Rhythmus-EKG, Sauerstoffsättigung, exspiratorisches CO_2, Blutdruck) und 12-Kanal-EKG erfolgt dabei nur durch dafür nach Medizinproduktegesetz zugelassene Systeme der Fa. Philips Healthcare (IntelliVue Information Center, Heartstart Telemedicine System). Zusätzlich zu diesen diagnostischen Instrumenten können übertragene Bilddateien und Videodateien angezeigt werden. Diese dienen ausdrücklich nicht der Diagnosefindung sondern nur zur Ergänzung der über die Sprachkommunikation ausgetauschten Gesprächsinhalte. Die Inhalte dieser Dateien müssen per

Sprachkommunikation verifiziert werden. Zudem stehen dem Telenotarzt Handlungsempfehlungen (SOP), Leitlinien und Medikamentendatenbanken EDV-basiert zur Verfügung. In der Telenotarzt-Zentrale erfolgt eine softwarebasierte, standardisierte Dokumentation aller telemedizinisch betreuten Einsätze.

Die Datenübertragung vom Rettungswagen bzw. der Einsatzstelle an die Telenotarzt-Zentrale sowie die bidirektionale Audioverbindung erfolgt stets verschlüsselt nach aktuellem Stand der Technik. Somit kann maximale Abhörsicherheit gewährleistet werden. Zur weiteren Datensicherheit werden alle Datenpakete mit einem elektronischen Schlüssel versehen. Bei Ankunft des Datenpaketes am Zielort (z.B. in der Telenotarzt-Zentrale) kann mit Hilfe dieses Schlüssels die Echtheit der Daten verifiziert werden. Korrekt und nicht-korrekt übermittelte Daten können so differenziert werden und nur korrekt übertragene Daten kommen zur Anzeige. Die Speicherung dieser einsatzbezogenen Daten erfolgt auf speziell abgesicherten Servern. Dabei werden Videodaten grundsätzlich nicht abgespeichert. Nur die medizinische Projektleitung hat Zugriff auf die einsatzbezogenen Daten.

4.2.2 Rettungswagen

Insgesamt sechs Rettungswagen aus den fünf oben erwähnten Rettungsdienstbereichen werden telemedizinisch ausgestattet. Dabei werden zwei Systeme ergänzend zum Einsatz gebracht. Im Fahrzeug selbst kommt ein stationäres Übertragungssystem (P3 communications, Aachen) mit vier parallelen Datenkanälen aller deutschen Mobilfunkanbieter zum Einsatz. Dies bietet maximale Sicherstellung der Verfügbarkeit. Im Projekt Med-on-@ix konnte gezeigt werden, dass durch die parallele Verwendung von vier Datenkanälen die Wahrscheinlichkeit der Verfügbarkeit eines Datendienstes von 95% auf 99% steigt, im Vergleich zu einer kommerziellen Standardlösung mit einer einzelnen UMTS-Mobilfunkkarte. Zum mobilen Einsatz an einer Einsatzstelle (z.B. Wohnung des Patienten) wird eine kompakte, tragbare Datenübertragungseinheit (P3 communications, Aachen) eingesetzt, die ca. 1kg wiegt. Hiermit sind „nur" zwei parallele Datenkanäle, jedoch aller vier deutschen Mobilfunkanbieter, zeitgleich zu nutzen. Dieses stellt einen Kompromiss dar, da die mobile Einheit leicht und energiesparend sein muss, um eine einsatztaktisch ausreichend lange Akkulaufzeit zu haben.

Beide Systeme bieten folgende Funktionalitäten:

- Audioverbindung zwischen Rettungsteam und Telenotarzt-Zentrale
- kontinuierliche Übertragung von Vitalparametern (EKG, Pulsoxymetrie, Blutdruck, endtidales CO_2 -Kurven und Zahlenwerte -) (IntelliVue Information Center, Philips Healthcare); Die Monitor-Defibrillator-Einheit Heartstart MRx (Philips Healthcare) ist mit der Übertragungseinheit via Ethernet und Bluetooth verbunden.
- periodische Übertragung von Vitalwerten im einminütigen Intervall (Heartstart Telemedicine System, Philips Healthcare)
- Versand eines 12-Kanal-EKGs via Telemedizinsystem (Heartstart Telemedicine System, Philips Healthcare) oder via Mobilfax
- Versand von Fotos, die mit einer Digitalkamera erzeugt werden

- Übertragung von Auskultationsgeräuschen eines elektronischen Stethoskops (Littmann 3200, 3M)
- im Rettungswagen besteht zusätzlich die Möglichkeit einer Videoübertragung mit Hilfe einer fest verbauten Deckenkamera (SNC-RZ 50P, Sony Electronics Inc, USA)

Wenn das mobile System im Fahrzeug konnektiert wird, erfolgt automatisch die Umschaltung auf das stationäre System.

4.2.3 Einbindung von Medizinprodukten

Die Monitor-Defibrillator-Einheit Heartstart MRx der Fa. Philips Healthcare wird verwendet. Mit Hilfe einer Ethernetschnittstelle und Bluetooth ist diese mit der mobilen oder stationären Übertragungseinheit konnektiert und überträgt die Daten automatisiert an die Telenotarzt-Zentrale. Ein Unterbrechen dieser Übertragung ist durch Ausschalten der Übertragungseinheit jederzeit möglich. Es finden keinerlei Eingriffe in das eigentliche Medizinprodukt statt, sondern dieses ist lediglich zu Anzeigezwecken von Vitalparametern durch dafür vom Hersteller vorgesehene Übertragungswege mit einer externen Stelle verbunden.

Die Anzeige dieser Daten erfolgt ausschließlich über dafür von Philips zur Verfügung gestellte Software, die nach Medizinproduktegesetz (MPG) zertifiziert sind (IntelliVue Information Center, Heartstart Telemedicine System, beides Philips Healthcare).

Die Fa. Philips ist von Beginn an der Entwicklung des mobilen Netzwerkes zur Übertragung beteiligt. Daher konnten alle Anforderungen, die nach MPG-Zertifizierung an dieses Netzwerk bestehen, berücksichtigt werden. Nach Fertigstellung der Entwicklung wird die Fa. Philips das Netzwerk überprüfen und ein Zertifikat ausstellen, dass alle vom Hersteller geforderten Standards erreicht wurden. Da die Fertigstellung des Systems für Ende Oktober 2011 geplant ist, wird dieses Zertifikat der Ethikkommission des Universitätsklinikums Aachen nachgereicht.

Ein elektronisches Stethoskop der Fa. 3M / Littmann wird mit Hilfe einer Bluetooth Schnittstelle ebenfalls mit den Übertragungseinheiten verbunden. Die Auskultationsgeräusche können in der Telenotarzt-Zentrale mit Hilfe eines zweiten elektronischen Stethoskops empfangen und gehört werden. Die dafür eingesetzte Software ist vom Hersteller explizit für diesen Zweck nach MPG zugelassen.

Die Verwendung von Medizinprodukten und Nicht-Medizinprodukten in einem modularen System erfordert die Betrachtung und letztlich Beachtung des Medizinproduktegesetzes und der Medizinproduktebetreiberverordnung. Daher wurde Herr Prof. Fehn (Köln) als Gutachter beauftragt, die medizinprodukterelevanten, rechtlichen Fragestellungen vorab zu begutachten. Er kommt zu dem Ergebnis, dass der Einsatz des in diesem Prüfplan beschriebenen Systems gesetzeskonform ist, wenn die Geräte gemäß ihren Zweckbestimmungen eingesetzt werden. Die definierten Zweckbestimmungen der Geräte und des Systems sowie das Gutachten befinden sich im Anhang zu diesem Prüfplan.

Alle anderen verwendeten Geräte (Digitalkamera, Deckenkamera) werden nicht zu Diagnosezwecken eingesetzt, sondern dienen als ergänzende Informationsquellen zur Sprachkommunikation. Jedoch erfolgt auch hier keinerlei Eingriff in die Produkte selber, sondern sie werden gemäß ihrer Bestimmung

eingesetzt. Das System ist ein insgesamt modulares System, welches nicht den Gebrach aller Funktionalitäten gleichzeitig erfordert. Im Gegenteil werden im Sinne der Datensparsamkeit nur die Daten übertragen, die im konkreten Fall medizinisch erforderlich sind.

4.2.4 Telekonsultation

Die Telekonsultation findet bei der überwiegenden Zahl der Einsätze zu Beginn zwischen den Rettungsassistenten vor Ort und einem Telenotarzt statt. Die Entscheidung, eine Telekonsultation durchzuführen obliegt dabei den Rettungsassistenten nach erfolgter Aufklärung des Patienten über diese Konsultation. In dieser Phase der Rettungsassistent-Arzt-Konsultation verfolgt die Telekonsultation das Ziel, ärztliche Expertise bereit zu stellen, in einer Einsatzphase, bei der (noch) kein Notarzt vor Ort ist. Fragen zu seltenen Erkrankungen und Medikamenteninteraktionen können beantwortet und Hilfestellung bei der Beurteilung des 12-Kanal-EKGs kann angeboten werden. Bei einigen Einsätzen führen Rettungsassistenten seit Jahren im Rahmen der sog. Notkompetenz ausgewählte ärztliche Maßnahmen wie i.v.-Zugang und Applikation ausgewählter Medikamente selbstständig durch. Diese Maßnahmen können nun ärztlich begleitet und überwacht werden. Im Fall einer Komplikation, kann der Telenotarzt medizinische Anweisungen geben, die Folgeschäden verhindern können. Im Falle deutlich verlängerter Eintreffzeiten eines Notarztes können durch den Telenotarzt auch Maßnahmen an die Rettungsassistenten delegiert werden, die bisher im Regelfall von diesen nicht ohne Arzt vor Ort durchgeführt werden. Um den Patienten vor Schäden durch eine zu spät eingeleitete Therapie zu schützen, können durch den Telenotarzt, nach individueller ärztlicher Entscheidung im konkreten Einsatz, solche Medikationen delegiert werden. Beispielsweise ist die Delegation von Analgetikaapplikationen an das Pflegepersonal im Krankenhaus üblich, im Rettungsdienst jedoch die Ausnahme. Schwere Schmerzzustände oder andere akut bedrohliche Zustände erfordern jedoch eine möglichst zeitnahe, korrekte Medikation. In einem Pilotprojekt im Rettungsdienst Mittelhessen wurde bei insgesamt 172 Patienten Morphin algorithmusbasiert durch Rettungsassistenten zur Analgesie bei Extremitätentrauma appliziert. Dazu erfolgte eine telefonische Freigabe und Delegation durch einen Notarzt. In keinem dieser Fälle wurde eine bedrohliche Komplikation beobachtet und das Schmerzniveau konnte signifikant gesenkt werden.[26] Im Gegensatz zur telefonischen Delegation von Analgetika im Krankenhaus und zur rein telefonischen Delegation im Rahmen des Pilotprojektes in Mittelhessen, überwacht der Telenotarzt im Projekt TemRas den Patienten kontinuierlich weiter (EKG, Pulsoxymetrie, Blutdruck, Sprechverbindung mit Rettungsassistenten) bis dieser einem Arzt zugeführt ist (Notarzt trifft ein / Ankunft im Krankenhaus). Daher wird von einer zusätzlich erhöhten Sicherheit für den Patienten ausgegangen. Im Vorgängerprojekt Med-on-@ix wurden die rechtlichen Fragestellungen bezüglich der telemedizinischen Delegation ärztlicher Leistungen durch Gutachten von Herrn Prof. Katzenmeier und Herrn Prof. Fehn bereits umfassend beantwortet und der Ethikkommission des UKA zur Begutachtung vorgestellt (EK 141/09). Beide Gutachter kommen zu dem Ergebnis, dass die telemedizinische Delegation ärztlicher Leistungen grundsätzlich rechtskonform ist. Zudem betonen sie, dass dabei die Maßnahmen an Fachpersonal, das speziell für Notfallsituationen geschult ist, delegiert werden. Beide Gutachten befinden sich im Anhang dieses Prüfplans.

4.2 Datenquellen

Folgende Datenquellen werden im Rahmen der wissenschaftlichen Evaluation verwendet:

- Notarzteinsatzprotokolle und Rettungsdienstprotokolle der teilnehmenden Rettungsdienste; aus diesen werden die Fälle identifiziert die detailliert ausgewertet werden (Einschlusskriterien)
- einsatzbezogene Zeiterfassung der Rettungsleitstelle zu Berechnung von Eintreff- und Versorgungszeiten
- Daten aus den Krankenhausinformationssystemen der Krankenhäuser, denen die eingeschlossenen Patienten zugeführt wurden: Diagnosen nach ICD 10, Zeitstempel diagnostischer und therapeutischer Verfahren (z.B. Computertomographie, Herzkatheteruntersuchung), Laborwerte, Schweregrad mit Hilfe fallbezogener Scoringsysteme (z.B. National Institutes of Health Stroke Scale beim Schlaganfall), Krankenhausverweildauer.
- Dokumentation des Telenotarztes und empfangene Datenpakete in der Telenotarzt-Zentrale (z.B. EKG, Blutdruck…)

Da es sich bei diesen Daten um klinische Routinedaten handelt, die nicht gesondert für diese Studie erhoben werden, ist von einer hohen Validität der Daten auszugehen. Zudem entsteht keinerlei Zusatzbelastung für das medizinische Personal und keine zusätzliche Erhebung und Speicherung von sensiblen, persönlichen Patientendaten.
Nachdem ein Patient in die Studie eingeschlossen wurde, werden die erforderlichen Daten durch die Prüfärzte aus den nicht-anonymisierten Datenquellen (z.B. Krankenhausinformationssystem) heraus gelesen und in eine anonymisierte Datenbank übertragen. Die eigentlichen statistischen Auswertungen, an der auch nicht-ärztliches Personal wie Biostatistiker beteiligt sind, erfolgen dann vollständig anonymisiert. Somit erfolgt die Datenerhebung zwar prospektiv, die Auswertung hingegen hat jedoch eher den Charakter einer retrospektiven, anonymisierten Auswertung.

Im Rahmen der Akzeptanzuntersuchungen werden anonymisierte Fragebögen sowohl von Rettungsassistenten, Notärzten und Telenotärzten ausgefüllt als auch anonymisierte Fragebögen an Patienten geschickt. Zudem erfolgt die Erhebung und anonymisierte Dokumentation qualitativen Feedbacks des Rettungsdienstpersonals im Rahmen von Gruppendiskussionen und Einsatzbesprechungen. Im Anhang sind die Fragebögen für Patienten und rettungsdienstliches Fachpersonal zu finden.

Zur Erhebung von Strukturdaten der Rettungsdienste erfolgen Gespräche mit Verantwortlichen im jeweiligen Rettungsdienstbereich sowie fragebogenbasierte Abfragen bei diesen Personen. Auch hier wird auf Daten der Rettungsleitstelle zurückgegriffen. Dies sind jedoch keine patientenbezogenen

Daten, sondern allgemeine statistische Daten wie z.B. „durchschnittliche Eintreffzeit“, „Notarztquote“, „Einsatzspektrum“.

4.3 Risiko-Nutzen-Abwägung und Vorsichtsmaßnahmen

Wenn die Versorgung von Notfallpatienten durch einen Telenotarzt unterstützt wird, ist insgesamt von einer Erhöhung der Patientensicherheit auszugehen. Das in TemRas verwendete System wird lediglich als zusätzliche Unterstützung eingesetzt und ersetzt nicht bestehende Systeme. Es kommt in den meisten Fällen in einer Einsatzphase zur Anwendung, in der die Rettungsassistenten zum jetzigen Zeitpunkt, keinen ärztlichen Rat zur Verfügung haben. Mit dem Einsatz der Telekonsultation kann diese Versorgungslücke geschlossen werden und ärztlicher Rat steht umgehend zu Verfügung, was im Einzelfall sogar lebensrettend sein kann. Daher überwiegt aus heutiger Sicht klar der Nutzen die potentiellen Risiken. Die Abwägung zwischen Nutzen und Risiken, die Untersuchung der Risiken und die Festlegung der zu treffenden Vorsichtsmaßnahmen werden vor und während der Studie im Rahmen eines kontinuierlichen Risikomanagementprozesses durchgeführt.

Folgende potentielle Risiken sind im Rahmen von Workshops unter Beteiligung aller Konsortialpartner identifiziert worden:

- Ablenkung des Personals vor Ort durch die Telekonsultation
- fehlgerichtete Kommunikation als Ursache für einen solchen Ablenkungseffekt
- Fehlentscheidungen, bei partiellem oder totalem Ausfall von Komponenten oder Datenverbindungen

Risikomanagementprozess

Ziel des Risikomanagementprozesses ist die Minimierung der Risiken des Systems für den Patienten indem drei wesentliche Eigenschaften des Netzwerks im Entwicklungsprozess sichergestellt werden: Sicherheit, Leistungsfähigkeit sowie Daten- und Systemschutz. Das Risikomanagement wird vom IMA der RWTH Aachen verantwortet und unter Einbeziehung der anderen Partner durchgeführt. Um die Risiken minimal zu halten, werden im Rahmen des Risikomanagementprozesses Risiken identifiziert und bewertet, Gegenmaßnahmen festgelegt und der Prozess in einer Risikomanagementakte dokumentiert.

Der Risikomanagementprozess orientiert sich an der DIN EN ISO 14971 (Abbildung 1). In der Risikoanalyse werden Risiken identifiziert und ihre Schwere und soweit möglich Eintrittswahrscheinlichkeit geschätzt. In der Risikobewertung wird betrachtet, ob Maßnahmen durchgeführt werden sollten. Falls ja, werden in der Risikobeherrschung Maßnahmen festgelegt, evaluiert und durchgeführt. Falls dadurch neue Risiken entstehen, müssen diese wieder evaluiert werden. Zum Schluss muss das Gesamtrestrisiko bewertet werden. Alle Phasen des Risikomanagementprozesses werden durch Workshops unterstützt. Besonderes Augenmerk liegt auf

einzelnen Komponentenausfällen, die für sich allein zum Ausfall des ganzen Systems führen könnten (Single-Points-of-Failure).

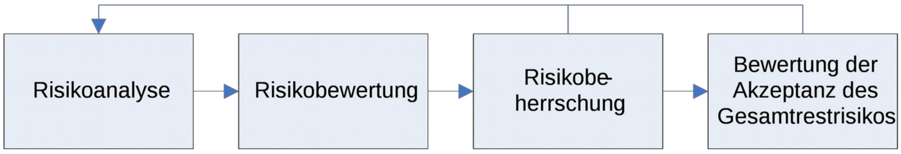

Abbildung 1 Risikomanagementprozess (vereinfacht)

Die Risikomanagementakte ist der zentrale Ort, an dem alle für das Risikomanagement relevanten Informationen gesammelt werden. Sie enthält Verweise auf die relevanten Entwicklungsdokumente (Anforderungen, Use-Cases, Standard Operating Procedures, BugTracker). Außerdem erfasst sie die Identifikation der Risiken und die dazu entschlossenen Gegenmaßnahmen.
Alle drei Monate finden Risikomanagement-Workshops statt, in denen im Sinn eines kontinuierlichen Verbesserungsprozesses der Stand der Umsetzung der Maßnahmen kontrolliert, die Maßnahmen auf ihre Effektivität überprüft und neue Risiken identifiziert werden.

Vorsichtsmaßnahmen

Zur Reduktion der Risiken im Umgang der beteiligten Akteure mit dem System werden diese vor Beginn der praktischen Evaluationsphase intensiv im Rahmen standardisierter Schulungen auf den Einsatz des Systems ausgebildet. Hierbei liegt der Fokus neben der Einweisung auf die Übertragungstechnik besonders auf der möglichst einheitlichen und zielgerichteten Kommunikation. Diese soll eine strukturierte, effiziente und sichere Übermittlung von Gesprächsinhalten ermöglichen. Angelehnt wird diese Kommunikation an die Kommunikation im Funk für Behörden und Organisationen mit Sicherheitsaufgaben (BOS-Funk, Rettungsdienst, Feuerwehr, Polizei), die den Beteiligten aus ihrem beruflichen Alltag bestens bekannt ist. In den Schulungen werden Fallbeispiele durchgeführt, die zum Ziel haben, genau diese Kommunikation zu trainieren. Weiterhin werden rechtliche Themen wie Aufklärung des Patienten, rechtliches Verhältnis Rettungsassistent-Telenotarzt und haftungsrechtliche Fragen in das Schulungskonzept integriert.
Als weitere Sicherheitsebene wird der Telenotarzt durch softwarebasierte Checklisten unterstützt, um trotz räumlicher Distanz zum Patienten, keine wesentlichen diagnostischen und therapeutischen Schritte zu übersehen. Die Vorteile einer checklistenbasierten Arbeitsweise und die positiven Einflüsse auf die Patientensicherheit sind aus dem WHO-Projekt „Safe Surgery Saves Lifes“ bestens bekannt. Sowohl für elektive als auch notfallmäßige Operationen konnte ein verbessertes Outcome durch checklistenbasiertes Arbeiten im „Hochrisiko-Bereich OP-Saal“ nachgewiesen werden.[21-23] Ein solches Checklistenprinzip zur Sicherstellung des notwendigen Mindeststandards ist auch auf die Telekonsultation übertragbar. In den SOP wird der Gebrauch dieser Checklisten für die Telenotärzte definiert. Beispielsweise darf die Delegation einer intravenösen Medikation erst dann erfolgen, wenn anhand der entsprechenden Checkliste alle notwendigen Informationen eingeholt (z.B. Allergien erfragt worden sind) und alle Sicherheitsmaßnahmen (z.B. Pulsoxymetrie, Blutdruckmessung,

Rhythmus-EKG) ergriffen sind. Im Projekt Med-on-@ix wurden bereits positive Erfahrungen mit checklistenbasierter Telekonsultation beim Schlaganfall gemacht. Durch Einsatz einer „Stroke Checkliste“ in der Telenotarzt-Zentrale konnte die Menge der schlaganfallspezifischen Informationen für den Neurologen in der Klinik mehr als verdoppelt werden ($p<0{,}0001$) (ergänzende Daten siehe Anlage). Die Erstellung von SOP und Checklisten erfolgt durch die Projektmitarbeiter nach wissenschaftlichen Kriterien. Dabei muss ein Dokument und von allen beteiligten Prüfärzten geprüft und von der Projektleitung freigegeben werden. Für Überarbeitungen dieser Dokumente innerhalb der Projektlaufzeit gilt das gleiche Verfahren.

Zur Analyse der Kommunikationsstandards und zur Sicherstellung eines hochwertigen Kommunikationsniveaus, finden in der Telenotarzt-Zentrale regelmäßig Supervisionen durch Kommunikationswissenschaftler des IMA/ZLW & IFU der RWTH Aachen statt. Diese haben zum Ziel ein konstruktives Feedback an die Telenotärzte zu geben, um eine weitere Optimierung der Kommunikation in der Projektlaufzeit zu erreichen. Zudem ist die Telenotarzt-Zentrale immer mit zwei Telenotärzten besetzt. Dies garantiert neben der erhöhten Verfügbarkeit auch ein regelmäßiges inter-kollegiales Feedback.

Der Telenotarzt beurteilt die Güte und Zuverlässigkeit der Datenverbindungen bei jeder Telekonsultation. Sollte aufgrund geringer Übertragungsraten eine kontinuierliche Übertragung von Vitalparametern nicht möglich sein, so darf er laut SOP keine Delegationen durchführen, die eine solche Überwachung sinnvoll erscheinen lassen. Jedoch muss an dieser Stelle bedacht werden, dass der Patient selbst bei vollständigem Funktionsausfall des gesamten Telemedizinsystems immer noch (wie aktuell deutschlandweit) durch qualifizierte Rettungsassistenten betreut wird, die in lebensrettenden ärztlichen Maßnahmen geschult und trainiert sind. Um bei Totalausfall des Systems trotzdem zumindest eine Sprachkommunikation zwischen Rettungsteam und Telenotarzt zu ermöglichen, verfügt jeder Rettungswagen zusätzlich über ein Mobiltelefon (Backup-Handy) wie es auch in der DIN EN 1789 für Rettungswagen gefordert wird. Die Telenotarzt-Zentrale verfügt zudem über einen handelsüblichen Festnetzanschluss, der selbst bei Ausfall der Internetverbindung erreichbar ist.

Im Rahmen eines IT-Sicherheitskonzepts werden die in TemRas anfallenden Daten vor unberechtigtem Zugriff, unberechtigter Veränderung und Verlust geschützt. Es beginnt mit einer Übersicht über das System und definiert den Prozess zur kontinuierlichen Überarbeitung des Konzepts. Die in TemRas anfallenden Patientendaten und die Geräte, die die Daten verarbeiten werden identifiziert und Schutzmechanismen definiert. Ferner wird festgehalten, wie lange die Daten nach Einsatzende aufbewahrt werden, wie sie geschützt werden und wer Zugang zu Ihnen hat.

Vor diesem Hintergrund überwiegen die Nutzen-Effekte eines solchen Systems. Maßnahmen zur Reduzierung möglicher Risiken werden umfassend ergriffen.

4.4 Unerwünschte Ereignisse

Unerwünschte Ereignisse können die Faktoren Technik, Organisation oder Medizin betreffen. Im Falle eines solches unerwünschten Ereignisses, das im Rahmen einer Telekonsultation auftritt, erfolgt eine standardisierte Dokumentation. Sowohl die Telenotärzte als auch das Rettungsfachpersonal verfügen dafür über Vorlagen. Diese Dokumentationen werden unverzüglich analysiert, um mögliche Probleme und Gefahrenquellen frühzeitig zu erfassen und kurzfristige Gegenmaßnahmen innerhalb des Projektzeiraums ergreifen zu können.

4.5 Interim Analysen

Monatlich werden umfassende Analysen der Dokumentationen der unerwünschten Ereignisse (falls angefallen) durchgeführt. Änderungen des Vorgehens und des Prüfplans erfolgen, falls notwendig, adaptiert an diese Ergebnisse.
Eine Zwischenauswertung von medizinischen Zielparametern ist aufgrund der Komplexität der Daten nicht vorgesehen. Zudem gibt es aus dem Vorgängerprojekt Med-on-@ix keinerlei Hinweise und Daten auf eine mögliche negative Beeinflussung des Behandlungsablaufs. Sollten sich jedoch aus der Dokumentationen der unerwünschten Ereignisse Hinweise ergeben, dass eine Zwischenauswertung notwendig ist, so wird eine solche unverzüglich durchgeführt.

4.6 Abbruch der Studie

4.6.1 Abbruch der Studie bei einem Probanden

Sollte es zu einem relevanten technischen Defekt im Einsatz kommen, so wird diese Telekonsultation abgebrochen. Es soll während einer Patientenversorgung kein Reparaturversuch am System unternommen werden. Nach dem Einsatz erfolgt dann die technische Diagnose und ggf. Reparaturmaßnahmen. Ein weiterer Grund wäre die sekundäre Ablehnung des Verfahrens durch Patienten, d.h. er zieht nach Beginn der Telekonsultation die Einwilligung in dieses Verfahren zurück.

4.6.2 Abbruch der gesamten Studie

Sollte es im Verlauf der Studie Anhaltspunkte dafür geben, dass es durch das Verfahren zu Schädigungen von Patienten kommt, so wird unverzüglich eine Interimsanalyse der bis dahin gewonnen Daten durchgeführt. Sollten sich solche Vermutungen bestätigen, würde dies zum Abbruch der Studie führen. Als mögliche Schädigungen seien genannt:

- signifikant schlechtere Reanimationsergebnisse
- signifikant verlängerte Versorgungszeiten bei lebensbedrohlichen, zeitkritischen Notfällen

- signifikant häufigere Komplikationen bei telemedizinischer Begleitung von Medikamentenapplikation durch Rettungsfachpersonal

5. Biometrie

5.1 Biometrisches Design

Die Studie wird als Prä-post-Interventionsstudie durchgeführt. Mit Start der praktischen Evaluationsphase beginnt eine prospektive Datenkollektion vom 01.08.2012 und endet geplanter Weise am 31.07.2013. Die Daten des Prä-Interventionszeitraums werden folgendermaßen analysiert: Die Definition der auszuwertenden Daten erfolgt analog zur Post-Interventionsphase prospektiv. Jedoch erfolgt die eigentliche Auswertung retrospektiv. Es handelt sich bei den Daten nur um klinische Routinedaten, nicht um gesondert für diese Studie erhobene Daten. Die statistische Auswertung erfolgt dann vollständig anonymisiert.

5.2 Stichprobenplanung

Planungsgröße ist die geschätzte Gesamtanzahl von 16 Rettungswageneinsätzen pro Studientag. Bei ca. 5,5 dieser Einsätze erfolgt der parallele Einsatz eines Notarztes. Nach lokaler Expertenmeinung ist daher von 5 bis 6 Konsultationen pro Tag auszugehen.

Bei 250 Arbeitstagen in der post-Interventions-Phase ergibt sich somit eine Anzahl von 1250 bis 1500 Konsultationen. Für die Krankheitsbilder Akutes Koronarsyndrom und den Schlaganfall liegen exakte Daten aus dem Vorgängerprojekt Med-on-@ix vor. Das akute Koronarsyndrom fand sich in 14,3% aller Notarzteinsätze und der Schlaganfall/ICB in 10% aller Notarzteinsätze als primäre Diagnose. Eine multizentrische deutsche Analyse aus dem Jahr 2005 bestätigt diese Häufigkeiten.[27]

Ausgehend von diesen Zahlen kann man von ungefähr 179 bis 215 Fällen von akutem Koronarsyndrom und 125 bis 150 Fällen von akutem Schlaganfall ausgehen. Für die anderen auszuwertenden Krankheitsbilder liegen keine so exakten Daten vor. Es ist bei diesen Notfällen mit einer Häufigkeit von ca. 5% pro Krankheitsbild zu rechnen (62-75 Fälle). Mit dem akuten Koronarsyndrom und dem Schlaganfall, stellen sie die häufigsten Notfallbilder dar.

5.3 Datenerfassung und Auswertung

Die Datenerfassung erfolgt wie oben beschrieben nur durch Prüfärzte des UKA. Die anonymisierte Datenauswertung erfolgt dann in Kooperation mit dem Institut für medizinische Statistik des UKA.

5.4 Statistische Methoden

Die primären und sekundären Outcomeparameter werden vor und nach der Intervention miteinander statistisch verglichen. Parametrische und nicht-parametrische statistische Verfahren kommen dabei zum Einsatz. Sollten sich mögliche Einflussfaktoren auf die Ergebnisse zeigen, so werden diese z.B. im Rahmen einer Varianzanalyse ausgewertet.

6. Änderungen des Prüfplans

Sollten sich im Verlauf der Studie notwendige Änderungen im Prüfplan ergeben, so wird unverzüglich die Ethikkommission des UKA darüber informiert und die Änderungen im Studienregister publiziert.

7. Ethische und rechtliche Belange

7.1 Rechtliche Grundlagen

- RettG NRW: Alle dort geforderten Standards werden nicht berührt.

- Rechtsgutachten: In den beiden Rechtsgutachten von Prof. Fehn und im Gutachten von Prof. Katzenmeier[28] wird dargestellt, dass die Durchführung der hier geplanten Studie rechtskonform möglich ist. Die Inhalte der Rechtsgutachten werden den Rettungsassistenten in kompakter Form und den Telenotärzten in detaillierter Form im Rahmen der Schulungen vermittelt. Die vollständigen Rechtsgutachten können von jedem Beteiligten eingesehen werden.

- Berufsordnung für Ärzte: Die Berufsordnung für Ärzte wird nicht negativ berührt.

- Es werden umfassende Maßnahmen zur Sicherung der höchst schützenswerten Patientendaten, wie im Bundesdatenschutzgesetz gefordert, ergriffen.

7.2 Votum der Ethikkommission

Da alle im Projekt in der Funktion als Telenotarzt eingesetzten Ärzte aus der Klinik für Anästhesiologie des Universitätsklinikum Aachen entstammen und die Telenotarzt-Zentrale auch durch diese Klinik betrieben wird, wird ein Ethikantrag bei der Ethikkommission des Universitätsklinikums Aachen eingereicht.
Es erfolgt kein Beginn der praktischen Studienphase (Post-Interventionsphase) mit Telekonsultation ohne ein positives Ethikvotum.

7.3 Leiter der klinischen Studie

Dr. med. Jörg Brokmann (Ärztlicher Leiter Rettungsdienst Stadt Aachen, Oberarzt der Klinik für Anästhesiologie des UKA)
Univ.-Prof. Dr. med. Rolf Rossaint (Direktor der Klinik für Anästhesiologie des UKA)

7.4 weitere Prüfärzte

PD Dr. med. Stefan Beckers (Oberarzt Operative Intensivmedizin UKA)
Dr. med. Michael Czaplik (Anästhesiologie UKA)
Dr. med. Sebastian Bergrath (Anästhesiologie UKA)
Dr. med. Harold Fischermann (Anästhesiologie UKA)
Dr. med. Frederik Hirsch (Anästhesiologie UKA)
Daniel Wielpütz (Anästhesiologie UKA)

7.5 Archivierung und Datenschutz

Alle Patientendaten und einsatztaktischen Daten, die zu wissenschaftlichen Auswertungszwecken aufbewahrt werden müssen, werden sicher verschlossen im Universitätsklinikum Aachen in der Klinik für Anästhesiologie aufbewahrt. Dabei wird möglichst auf die Speicherung und Archivierung von identifizierbaren Merkmalen verzichtet sondern der pseudonymisierten und anonymsierten Form Vorrang gegeben. Nach der wissenschafltichen Auswertung, werden identifizierbare Daten vernichtet und lediglich die pseudonymisierten und anonymisierten Daten aufbewahrt.
Alle regulären Patientendaten, die zu medizinischen Dokumentationszwecken archiviert werden, werden wie oben dargestellt, besonders gesichert auf Servern gespeichert.

7.6 Versicherung der ärztlichen Tätigkeit als Telenotarzt

hier einfügen: Patientenversicherung etc

8. Publikation

Für die unter 2.2 aufgeführten Krankheitsbilder sollen die Ergebnisse in medizinischen Fachzeitschriften veröffentlicht werden. Dabei werden englischsprachige Journals angestrebt. Die Akzeptanzuntersuchungen sollen ebenfalls veröffentlicht werden. Hierbei sind sowohl deutschsprachige als auch englischsprachige Publikationen angestrebt.

9. Zusammenfassung

Die telemedizinische Vernetzung zwischen medizinischem Personal und Spezialisten hat sich in vielen Bereichen der Medizin als vorteilhaft erwiesen. In der Notfall- und Akutmedizin kommen beispielsweise Telemedizinsysteme zwischen Krankenhäusern beim Schlaganfall zur Anwendung. Die Qualität der Patientenversorgung und das Outcome der Patienten konnten dadurch verbessert werden. In der präklinischen Notfallrettung kommt regelmäßig lediglich die Übertragung eines 12-Kanal-EKG an einen Kardiologen zum Einsatz. Für Patienten mit Myokardinfarkt konnten dadurch Versorgungszeiten verkürzt werden und letztlich das Outcome der Patienten verbessert werden. Andere telemedizinische Anwendungen kommen, abgesehen von zwei deutschen Pilotprojekten, nicht zum Einsatz. Der deutsche Rettungsdienst steht aktuell vor großen Herausforderungen. Speziell die Ressource „Notarzt" ist in manchen Regionen nicht mehr flächendeckend verfügbar. Zudem hat die zunehmende Auslastung der Notarztstandorte durch stetig steigende Einsatzzahlen bereits zu merklich verlängerten Eintreffzeiten des Notarztes beim Patienten geführt. In aller Regel trifft ein Rettungswagen, besetzt mit qualifizierten Rettungsassistenten, bereits mehrere Minuten vor dem Notarzt beim Patienten ein, da das Standortnetz der Rettungswagen deutlich dichter ist als das der Notarztstandorte. In dieser Phase, in der (noch) kein Notarzt vor Ort ist, übernehmen die Rettungsassistenten eigenverantwortlich die medizinische Versorgung. Regelmäßig applizieren sie auch ausgewählte Medikamente und ergreifen ausgewählte invasive Maßnahmen vor Eintreffen des Notarztes.

Um diese potentielle Versorgungslücke zwischen Eintreffen des Rettungswagen und Eintreffen des Notarztes zu minimieren, wird im Projekt TemRas (telemedizinisches Rettungsassistenzsystem) ein modulares, präklinisches Telemedizinsystem in einer einjährigen Evaluationsphase auf sechs Rettungswagen eingesetzt. Das System ermöglicht die Datenübertragung von Vitalparametern, 12-Kanal-EKGs, Auskultationsgeräuschen, Bildern und ggf. Videos an eine sog. Telenotarzt-Zentrale, die mit erfahrenen Notärzten (Telenotärzte) besetzt ist. Zu Diagnosezwecken verwendete Daten, werden nur über nach Medizinproduktegesetz zertifizierte Systeme, versendet. Im Rahmen dieser Telekonsultation wird eine Sprechverbindung zwischen den Rettungsassistenten und einem Telenotarzt aufgebaut. Der Telenotarzt kann das Team vor Ort in medizinischen und organisatorischen

Fragen beraten. Zudem wird die Durchführung ärztlicher Maßnahmen zeitgleich überwacht. In ausgewählten Fällen kann über ein solches System auch die Delegation ärztlicher Maßnahmen erfolgen, wie beispielsweise die Delegation der Applikation von Analgetika vor Eintreffen eines Notarztes, um schwere Schmerzen frühzeitig zu lindern. Die Entscheidung ob eine Telekonsultation durchgeführt wird, obliegt grundsätzlich den Rettungsassistenten vor Ort. Wenn diese feststellen, dass ärztlicher Rat erforderlich ist, können sie die Telekonsultation durchführen.

Die klinische, praktische Evaluation des Systems soll vom 01.08.2012 bis 31.07.2013 wochentags in der Zeit von 7.30 bis 16.30 Uhr im Rahmen einer Prä-post-Interventionsstudie erfolgen. Vor Einführung des Systems nehmen das Rettungsfachpersonal und die Telenotärzte an einem standardisierten Schulungskonzept teil. Es werden im Rahmen der praktischen Evaluationsphase (Post-Interventionsphase) prospektiv definierte Outcomeparameter aus klinischen Routinedaten analysiert. Es erfolgt anschließend der Vergleich mit einem einjährigen Zeitraum vor Schulung und Einführung des Systems. Die Daten dieses Prä-Interventionszeitraums werden retrospektiv und anonymisiert ausgewertet.

10. Literatur

1. Demaerschalk BM, Bobrow BJ, Raman R, et al. Stroke team remote evaluation using a digital observation camera in Arizona: the initial mayo clinic experience trial. *Stroke* 2010;**41**:1251-8.
2. Audebert HJ, Schenkel J, Heuschmann PU, Bogdahn U, Haberl RL. Effects of the implementation of a telemedical stroke network: the Telemedic Pilot Project for Integrative Stroke Care (TEMPiS) in Bavaria, Germany. *Lancet Neurol* 2006;**5**:742-8.
3. Audebert HJ, Kukla C, Clarmann von Claranau S, et al. Telemedicine for safe and extended use of thrombolysis in stroke: the Telemedic Pilot Project for Integrative Stroke Care (TEMPiS) in Bavaria. *Stroke* 2005;**36**:287-91.
4. Meyer BC, Raman R, Hemmen T, et al. Efficacy of site-independent telemedicine in the STRokE DOC trial: a randomised, blinded, prospective study. *Lancet Neurol* 2008;**7**:787-95.
5. Audebert HJ, Schultes K, Tietz V, et al. Long-term effects of specialized stroke care with telemedicine support in community hospitals on behalf of the Telemedical Project for Integrative Stroke Care (TEMPiS). *Stroke* 2009;**40**:902-8.
6. Sejersten M, Sillesen M, Hansen PR, et al. Effect on treatment delay of prehospital teletransmission of 12-lead electrocardiogram to a cardiologist for immediate triage and direct referral of patients with ST-segment elevation acute myocardial infarction to primary percutaneous coronary intervention. *Am J.Cardiol.* 2008;**101**:941-46.
7. Terkelsen CJ, Norgaard BL, Lassen JF, et al. Telemedicine used for remote prehospital diagnosing in patients suspected of acute myocardial infarction. *J Intern Med* 2002;**252**:412-20.
8. Dhruva VN, Abdelhadi SI, Anis A, et al. ST-Segment Analysis Using Wireless Technology in Acute Myocardial Infarction (STAT-MI) trial. *J.Am Coll.Cardiol.* 2007;**50**:509-13.
9. Adams GL, Campbell PT, Adams JM, et al. Effectiveness of prehospital wireless transmission of electrocardiograms to a cardiologist via hand-held device for patients with acute myocardial infarction (from the Timely Intervention in Myocardial Emergency, NorthEast Experience [TIME-NE]). *Am J.Cardiol.* 2006;**98**:1160-64.
10. Sanchez-Ross M, Oghlakian G, Maher J, et al. The STAT-MI (ST-Segment Analysis Using Wireless Technology in Acute Myocardial Infarction) trial improves outcomes. *JACC Cardiovasc Interv* 2011;**4**:222-7.

11. Ziegler V, Rashid A, Muller-Gorchs M, et al. [Mobile computing systems in preclinical care of stroke. Results of the Stroke Angel initiative within the BMBF project PerCoMed]. *Anaesthesist* 2008;**57**:677-85.
12. Skorning M, Bergrath S, Rortgen D, et al. [E-health in emergency medicine - the Research project Med-on-@ix.]. *Anaesthesist* 2009.
13. Schwamm LH, Holloway RG, Amarenco P, et al. A review of the evidence for the use of telemedicine within stroke systems of care: a scientific statement from the American Heart Association/American Stroke Association. *Stroke* 2009;**40**:2616-34.
14. Schwamm LH, Audebert HJ, Amarenco P, et al. Recommendations for the implementation of telemedicine within stroke systems of care: a policy statement from the American Heart Association. *Stroke* 2009;**40**:2635-60.
15. Ting HH, Krumholz HM, Bradley EH, et al. Implementation and integration of prehospital ECGs into systems of care for acute coronary syndrome: a scientific statement from the American Heart Association Interdisciplinary Council on Quality of Care and Outcomes Research, Emergency Cardiovascular Care Committee, Council on Cardiovascular Nursing, and Council on Clinical Cardiology. *Circulation* 2008;**118**:1066-79.
16. Bergrath S, Rörtgen D, Rossaint R, et al. Technical and organisational feasibility of a multifunctional telemedicine system in an emergency medical service - an observational study. *J Telemed Telecare* 2011 (epub ahead of print)
17. Behrendt H SR. Time comparison of current performance by emergency medical services in the Federal Republic of Germany (part II). *German Interdisciplinary Journal of Emergency Medicine* 2004;**7**:59-70.
18. Reimann B, Maier BC, Lott R, Konrad F. Gef„hrdung der Notarztversorgung im l„ndlichen Gebiet. *Notfall & Rettungsmedizin* 2004;**7**:200-04.
19. Luiz T, van Lengen RH, Wickenkamp A, Kranz T, Madler C. [Operational availability of ground-based emergency medical services in Rheinland-Palatinate : State-wide web-based system for collation, display and analysis.]. *Anaesthesist* 2011
20. Kollmann. Die Akzeptanz innovativer Nutzungsgüter und -systeme:
Konsequenzen für die Einführung von Telekommunikations- und Multimediasystemen. *Wiesbaden* 1998.
21. Weiser TG, Haynes AB, Dziekan G, et al. Effect of a 19-item surgical safety checklist during urgent operations in a global patient population. *Ann Surg*;**251**:976-80.
22. Haynes AB, Weiser TG, Berry WR, et al. A surgical safety checklist to reduce morbidity and mortality in a global population. *N Engl J Med* 2009;**360**:491-9.
23. Haynes AB, Weiser TG, Berry WR, et al. Changes in safety attitude and relationship to decreased postoperative morbidity and mortality following implementation of a checklist-based surgical safety intervention. *BMJ Qual Saf*;**20**:102-7.
24. Olasveengen TM, Sunde K, Brunborg C, et al. Intravenous drug administration during out-of-hospital cardiac arrest: a randomized trial. *JAMA* 2009;**302**:2222-9.
25. Bottiger BW, Arntz HR, Chamberlain DA, et al. Thrombolysis during resuscitation for out-of-hospital cardiac arrest. *N Engl J Med* 2008;**359**:2651-62.
26. Greb I, Wranze E, Hartmann H, Wulf H, Kill C. Analgesie beim Extremitätentrauma durch Rettungsfachpersonal. *Notfall & Rettungsmedizin* 2011;**14**:135-42.
27. Gries A, Zink W, Bernhard M, Messelken M, Schlechtriemen T. Einsatzrealität im Notarztdienst. *Notfall & Rettungsmedizin* 2005;**8**:391-98.
28. Katzenmeier C, Schrag-Slavu S. Rechtsfragen des Einsatzes der Telemedizin im Rettungsdienst. *Springer, Berlin Heidelberg* 2010.

11. Anlagen

11.1 Übersicht Studien

11.2 Zweckbestimmung des Systems und der verwendeten Geräte

11.3 CE-Kennzeichnung von verwendeten Geräten

11.4 Rechtsgutachten TemRas Prof. Fehn 2011

11.5 Rechtsgutachten Med-on-@ix Prof. Katzenmeier 2009

11.6 Rechtsgutachten Med-on-@ix Prof. Fehn 2009

11.7 Ergebnisse Projekt Med-on-@ix (bisher nicht publizierte Ergebnisse)

11.8 Fragebögen für Patienten und Rettungdienstpersonal

Aachen, XX.10.2011

Dr. med. Jörg Brokmann

Univ.-Prof. Dr. med. Rolf Rossaint
