# Supplementary material for: Treatment of Acute Coronary Syndrome by Telemedically Supported Paramedics Compared With Physician-Based Treatment: A Prospective, Interventional, Multicenter Trial
Source: J Med Internet Res. 2016 Dec 1;18(12):e314. doi: 10.2196/jmir.6358 (PMC5159613; doi:10.2196/jmir.6358)
Supplement: Supplementary file 3 [file jmir_v18i12e314_app3.pdf]

Datensatz: *Tabelle_MTamm.xlsx* (vom 19.11.2014)

Auswertungsmethode: Kontingenztafel für Matched-Pairs,
Exakter McNemar-Test (da Matched-Pair-Analyse)
(das bedeutet, dass die zusammengehörigen Paare immer direkt miteinander verglichen werden, Wichtig: Matching-Kriterien angeben)

Hinweis: Nicht signifikant bedeutet nicht, dass nachgewiesen wurde, dass die Methoden gleich gut sind!

**Table of _2kanal_temras by _2kanal_match**			
**_2kanal_temras**	**_2kanal_match**		
**FrequencyPercentRow PctCol Pct**	**0**	**1**	**Total**
**0**	00.000.00.	12.56100.002.56	12.56
**1**	00.000.00.	3897.44100.0097.44	3897.44
**Total**	00.00	39100.00	39100.00

Statistics for Table of _2kanal_temras by _2kanal_match

**McNemar's Test**	
**Statistic (S)**	1.0000
**DF**	1
**Asymptotic Pr > S**	0.3173
**Exact Pr >= S**	1.0000

Table of ASS_TemRas by ASS _Match			
ASS_TemRas(ASS_TemRas)	ASS_Match(ASS_Match)		
FrequencyPercentRow Pct Col Pct	0	1	Total
0	25.2628.5740.00	513.1671.4315.15	718.42
1	37.899.6860.00	2873.6890.3284.85	3181.58
Total	513.16	3386.84	38100.00
Frequency Missing = 1			

Statistics for Table of ASS_TemRas by ASS_Match

McNemar's Test	
Statistic (S)	0.5000
DF	1
Asymptotic Pr > S	0.4795
Exact Pr >= S	0.7266

Effective Sample Size = 38 Frequency Missing = 1

Table of Heparin_TemRas by Heparin_Match			

Heparin_TemRas(Heparin_TemRas)	Heparin_Match(Heparin_Match)		
FrequencyPercentRow PctCol Pct	0	1	Total
0	00.000.000.00	410.53100.0012.12	410.53
1	513.1614.71100.00	2976.3285.2987.88	3489.47
Total	513.16	3386.84	38100.00
Frequency Missing = 1			

Statistics for Table of Heparin_TemRas by Heparin_Match

McNemar's Test	
Statistic (S)	0.1111
DF	1
Asymptotic Pr > S	0.7389
Exact Pr >= S	1.0000

Effective Sample Size = 38 Frequency Missing = 1

Table of Morphin_TemRas by Morphin_Match			
Morphin_TemRas(Morphin_TemRas)	Morphin_Match(Morphin_Match)		
FrequencyPercentRow PctCol Pct	0	1	Total

0	00.00.0.00	00.00.0.00	00.00
1	26.906.90100.00	2793.1093.10100.00	29100.00
Total	26.90	2793.10	29100.00
Frequency Missing = 10			

Statistics for Table of Morphin_TemRas by Morphin_Match

McNemar's Test	
Statistic (S)	2.0000
DF	1
Asymptotic Pr > S	0.1573
Exact Pr >= S	0.5000

Effective Sample Size = 29 Frequency Missing = 10

Table of O2_TemRas by O2_Match			
O2_TemRas(O2_TemRas)	O2_Match(O2_Match)		
FrequencyPercentRow Pct Col Pct	0	1	Total
0	38.8260.0018.75	25.8840.0011.11	514.71
1	1338.2444.8381.25	1647.0655.1788.89	2985.29
Total	1647.06	1852.94	34100.00

Frequency Missing = 5			

Statistics for Table of O2_TemRas by O2_Match

McNemar's Test	
**Statistic (S)**	8.0667
**DF**	1
**Asymptotic Pr > S**	0.0045
**Exact Pr >= S**	0.0074

Effective Sample Size = 34 Frequency Missing = 5

WARNING: 13% of the data are missing.
