# Supplementary material for: Treatment of Acute Coronary Syndrome by Telemedically Supported Paramedics Compared With Physician-Based Treatment: A Prospective, Interventional, Multicenter Trial
Source: J Med Internet Res. 2016 Dec 1;18(12):e314. doi: 10.2196/jmir.6358 (PMC5159613; doi:10.2196/jmir.6358)
Supplement: Supplementary file 4 [file jmir_v18i12e314_app4.pdf]

Demographic Data

Age in years		
Minimum	31.00	34.00
Maximum	89.00	94.00
Mean	68.05	67.72
Std. Deviation	14.81	15.09
Std. Error	2.372	2.416
	P = 0,7393	
Table Analyzed	*Paired t test data*	
Wilcoxon matched-pairs signed rank test		
Exact or approximate P value?	*Exact*	
Significantly different? (P < 0.05)	*No*	
One- or two-tailed P value?	*Two-tailed*	
