# Supplementary material for: Treatment of Acute Coronary Syndrome by Telemedically Supported Paramedics Compared With Physician-Based Treatment: A Prospective, Interventional, Multicenter Trial
Source: J Med Internet Res. 2016 Dec 1;18(12):e314. doi: 10.2196/jmir.6358 (PMC5159613; doi:10.2196/jmir.6358)
Supplement: Supplementary file 5 [file jmir_v18i12e314_app5.pdf]

Detail table: correct handling of measures in each participating district

	Aachen (n=18)										
	Study group					Control group					
	correct		incorrect		no documentation	correct		incorrect		no documentation	
	given	not given	given	not given		given	not given	given	not given		
ASA[a]	12			6		16			2		ASA
ECG[b]	18					18					ECG
UFH[c]	12	3		3		17		1			UFH
morphine	3	10			5	8	3	1	1	5	morphine
oxygen	2	13	1		2	4	4	8		2	oxygen

	Dueren (n=3)										
	Study group					Control group					
	correct		incorrect		no documentation	correct		incorrect		no documentation	
	given	not given	given	not given		given	not given	given	not given		
ASA	1			1	1	3					ASA
ECG	2				1	3					ECG
UFH	3					2			1		UFH
morphine	1				2	1	1			1	morphine
oxygen		1	1		1			2		1	oxygen

	Euskirchen (n=12)										
	Study group					Control group					
	correct		incorrect		no documentation	correct		incorrect		no documentation	
	given	not given	given	not given		given	not given	given	not given		
ASA	12					9			2	1	ASA
ECG	12					12					ECG
UFH	9	2	1			9	1	1		1	UFH
morphine	2	9		1		3	7			2	morphine
oxygen		10	2			4	2	5		1	oxygen

	Heinsberg (n=6)										
	Study group					Control group					
	correct		incorrect		no documentation	correct		incorrect		no documentation	
	given	not given	given	not given		given	not given	given	not given		
ASA	6					5			1		ASA
ECG	6					6					ECG
UFH	4	1		1		4		1	1		UFH
morphine	2	2			2	1	3			2	morphine
oxygen	2	1	1		2	4		2			oxygen

	Overall (n=39)										
	Study group					Control group					
	correct		incorrect		no documentation	correct		incorrect		no documentation	
	given	not given	given	not given		given	not given	given	not given		
ASA	31			7	1	33			5	1	ASA
ECG	38				1	39					ECG
UFH	28	6	1	4		32	1	3	2	1	UFH
morphine	8	21		1	9	13	14	1	1	10	morphine
oxygen	4	25	5		5	12	6	17		4	oxygen

[a] ASA = acetylsalicylic acid

[b] ECG = electrocardiogram

[c] UHF = unfractionated heparin
